# Enhanced Bioavailability and Health Benefits of Blueberry Anthocyanins: An Updated Review on Mechanisms and Approaches

**DOI:** 10.3390/molecules31050793

**Published:** 2026-02-27

**Authors:** Rabia Ramzan, Zafarullah Muhammad, Adnan Amjad, Hafiz Rizwan Sharif, Guoqiang Zhang, Ana Chen

**Affiliations:** 1 College of Biological and Food Engineering, Anhui Polytechnic University, Wuhu 241000, China; rabiaramzan@mail.ahpu.edu.cn (R.R.);; 2 College of Food Science and Technology, Huazhong Agricultural University, Wuhan 430070, China; 3 College of Agriculture and Food Engineering, Baise University, Baise 533000, China; 4 Faculty of Food Science and Nutrition, Bahauddin Zakariya University, Multan 60000, Pakistan; 5 Institute of Food Science and Nutrition, University of Sargodha, Sargodha 40100, Pakistan

**Keywords:** blueberry anthocyanins, health potentials, bioavailability mechanism, influencing factors, stability improvement

## Abstract

Blueberries are highly valued for their nutritional content, primarily due to their high anthocyanin content, which is the principal bioactive compound contributing to their health-enhancing properties. Extensive research has established that blueberry anthocyanins exhibit significant antioxidant activity and confer various health benefits, including anti-cardiovascular, anti-diabetic, anti-inflammatory, and anticancer effects, as well as enhancements in cognitive function and visual acuity. Nonetheless, the chemical instability of anthocyanins, influenced by environmental factors such as temperature, light, oxygen, pH, and enzymatic activity, presents a substantial challenge for their effective application, leading to reduced stability, limited bioavailability, and decreased efficacy in functional foods and nutraceuticals. Using a defined search strategy focused on recent advances, this review synthesizes the chemical structures, biological activities, and health benefits of blueberry anthocyanins and critically examines strategies to improve their stability and bioavailability, including nanoparticulate systems, microencapsulation, based on delivery systems like protein, polysaccharides, liposomes, multiple emulsions, and composite delivery systems. Additionally, this review underscores the current research status and translational prospects for the industrial application of blueberry anthocyanins while also considering key scalability issues, including carrier regulation, sensory effects, and shelf-life stability. Developing practical approaches to enhance anthocyanin stability and bioavailability is crucial for maximizing their therapeutic potential and advancing the use of blueberries as functional foods.

## 1. Introduction

Blueberries (*Vaccinium* spp.) rank among the most favored berries globally because of their sweet and tangy taste and abundant nutritional attributes. They are esteemed for their phenolic content, especially flavonoids, chlorogenic acids, and anthocyanins, which confer distinct color and bioactivity [1]. Anthocyanins, a type of flavonoid, are pigments that impart red, purple, and blue colors to fruits, vegetables, and flowers. These compounds are formed when anthocyanidins bond with sugar molecules through glycosidic bonds, whereas anthocyanidins are their aglycone forms [2].

Among the anthocyanins found in nature, cyanidin-3-glucoside, delphinidin, malvidin, petunidin, and peonidin derivatives are the most prevalent in blueberries, with variations influenced by plant variety, environmental factors, and ripening stages [3,4]. Over 700 anthocyanins and 27 anthocyanidins have been identified in various plant species, highlighting the structural diversity of this compound class [4,5]. Anthocyanins are delicate molecules whose stability is affected by temperature, pH, light, oxygen, metal ions, and ascorbic acid [6]. In water-based solutions, these compounds are unstable at neutral or alkaline pH levels, leading to color loss and reduced bioactivity via chemical degradation. Despite this vulnerability, anthocyanins have garnered significant research attention because of their health benefits and potential use as natural colorants in the food industry [7]. Anthocyanins serve multiple physiological roles in plants. They attract pollinators and seed dispersers, thereby boosting reproductive success [8]. They protect against ultraviolet (UV) radiation and help mitigate oxidative stress by neutralizing reactive oxygen species (ROS) during environmental stress [9,10,11]. The antioxidant benefits observed in plants are similar to those in human health, making anthocyanins significant in functional food research.

Blueberry anthocyanins have been studied extensively for their antioxidant, anti-inflammatory, blood sugar-lowering, liver-protecting, and anticancer properties. Their antioxidant effects stem from their ability to neutralize reactive oxygen species (ROS) and protect cellular components from oxidative damage [12]. Oxidative stress contributes to various chronic illnesses, including cardiovascular diseases, neurodegenerative conditions, diabetes, and cancer. In vitro studies have shown that blueberry anthocyanins suppress cancer cell proliferation, trigger apoptosis, and influence signaling pathways related to inflammation and oxidative stress [9,13]. Anthocyanins decrease the expression of inflammatory cytokines, such as TNF-α and IL-6, which are associated with chronic inflammation [14,15]. Animal studies have demonstrated the protective properties of blueberry anthocyanins. Animal models have shown benefits in liver protection, neuroprotection, and the regulation of gut microbiota [16].

The scientific focus on blueberry anthocyanins has increased owing to their role in disease prevention. Studies have shown that anthocyanin-rich diets are correlated with a lower risk of cardiovascular diseases, type 2 diabetes, obesity, and neurodegenerative conditions [17,18]. Their antioxidant and anti-inflammatory properties may help alleviate chronic diseases linked to oxidative stress and inflammation. These findings highlight the importance of blueberries as a nutrient-rich food with health benefits. Anthocyanins decrease lipid peroxidation in hippocampal tissues, enhance cognitive abilities, and produce antidepressant-like effects in mice. Their microbiota modulating potential is significant, as they remain intact upon reaching the colon, where they interact with the gut microbiota, promoting beneficial bacterial growth and creating bioactive metabolites [19]. Human studies have demonstrated the functional benefits of blueberry consumption. Blueberry supplementation enhances cognitive abilities in children and adults [20,21], highlighting the neuroprotective properties of anthocyanins. Anthocyanin-rich blueberry products enhance cardiovascular indicators, manage glucose metabolism, and provide anti-inflammatory benefits in clinical settings, demonstrating their potential as functional dietary components [17,22].

Although blueberry anthocyanins offer many health benefits, their bioavailability is a challenge. Once consumed, anthocyanins undergo deglycosylation in the small intestine, releasing anthocyanidins, which are absorbed and metabolized by intestinal and liver enzymes [23]. Most anthocyanins are not absorbed in the upper gastrointestinal tract and reach the colon only. The gut microbiota breaks down these compounds into smaller phenolic compounds with bioactive properties. The stability, absorption, and metabolism of anthocyanins depend on their chemical structure, glycosylation pattern, and gastrointestinal conditions [24]. Factors such as food matrix interactions, pH levels, digestive enzymes, and bile salts impact anthocyanin bio-accessibility. To address these issues, strategies have been explored to enhance the stability and bioavailability of blueberry anthocyanins. Techniques such as microencapsulation, nanoencapsulation, and complexation with proteins, polysaccharides, lipids and multiple emulsion systems can protect anthocyanins from degradation and improve their gastrointestinal tract delivery [25,26]. These approaches maintain the functional properties of anthocyanins and expand their use in functional foods, nutraceuticals, and pharmaceuticals. The health benefits of blueberry anthocyanins depend on their stability, bioavailability, structural variety, and food component interactions. Sunlight, irrigation, and soil composition during growth influence the anthocyanin content and profile [25,27]. Postharvest processing, storage, and extraction methods affect the stability and bioactivities of these compounds. Understanding the composition, stability, metabolism, and bioavailability of anthocyanins is essential for harnessing the nutritional benefits of blueberries.

Numerous insightful reviews have explored various facets of the health benefits of blueberry anthocyanins. Herrera-Balandrano et al. [28] provided a detailed overview of strategies to enhance bioavailability, particularly through encapsulation methods. Yang et al. [29] conducted a systematic review of the structural diversity and functional attributes of blueberry anthocyanins, with a focus on antioxidant mechanisms. Silva et al. [17] compiled epidemiological and clinical evidence related to disease prevention, especially concerning cardiometabolic health. Kalt et al. [12] summarized recent research on health benefits across multiple areas. Wang et al. [23] recently examined stability challenges and food applications. Wu et al. [30] concentrated on health benefits and underlying mechanisms in preclinical models. Despite these valuable contributions, a significant gap persists, as no recent review has integrated structural, mechanistic, and technological perspectives with a quantitative and evidence-weighted synthesis of delivery system performance. Furthermore, previous reviews have not systematically compared delivery platforms using consistent parameters (particle size, encapsulation efficiency, release kinetics, and in vivo relevance) or explicitly linked anthocyanin structures to molecular pathways in a clinically relevant framework. To address these gaps, this review offers several distinct advances (Table 1). (i) A mechanistic, pathway-focused analysis explicitly linking specific anthocyanin structures (e.g., malvidin glycosides and delphinidin derivatives) to molecular targets (NF-κB, Nrf2, and MAPK) across health domains (neuroprotection, inflammation, cancer, and cardiometabolic health). (ii) A systematic, parameter-driven evaluation of delivery systems (Table 5) that quantitatively compares particle size, zeta potential, encapsulation efficiency, release kinetics, and in vitro/in vivo performance, an approach that has not been previously applied to blueberry anthocyanins. (iii) An integrated ADME-metabolite framework (Figure 5) emphasizing the role of gut microbiota-derived phenolic acids as key bioactive effectors, with a critical discussion of the parent compound vs. metabolite activity debate. (iv) A comprehensive synthesis of human clinical evidence (Table S1) with a critical appraisal of the study design, dosage, and outcomes. (v) A critical discussion of industrial translation challenges (scalability, regulatory status, sensory impact, shelf-life stability) that bridges laboratory research and commercial application. (vi) Identification of critical knowledge gaps and future research priorities to guide the field. By synthesizing recent advances (2018–2025) with this evidence-weighted comparative lens, this review provides researchers, nutritionists, and food technologists with a practical guide for designing next-generation anthocyanin-enriched functional foods and nutraceuticals. A comparative summary of recent reviews and the unique contributions of the present study is provided in Table 1.

## 2. Methodology

This narrative review provides a comprehensive and current overview of the mechanisms, health benefits, and methods to improve the bioavailability of blueberry anthocyanins. To maintain rigor and transparency, we used a structured search method in line with established scoping review guidelines [31], clearly outlining the search strategy, screening process, and data synthesis methodology.

### 2.1. The Strategy to Search Literature

On October 15, 2025, a comprehensive search was performed across three prominent scientific databases: PubMed/MEDLINE, Web of Science, and Scopus. This search focused on peer-reviewed articles published in English from January 2018 to October 2025, with the selective inclusion of key older works (pre-2018) to provide foundational context when necessary. Boolean operators (AND, OR) were used to combine the following primary terms and their variations in the search strategy:

(1) Population/Compound: (“blueberry” OR “blueberries” OR “Vaccinium” OR “Vaccinium corymbosum” OR “Vaccinium angustifolium” OR “Vaccinium ashei”). (2) Intervention/Component: (“anthocyanin” OR “anthocyanidin” OR “cyanidin” OR “delphinidin” OR “malvidin” OR “petunidin” OR “peonidin” OR “pelargonidin”). (3) Outcomes/Mechanisms: (“bioavailability” OR “absorption” OR “metabolism” OR “pharmacokinetics” OR “ADME” OR “stability” OR “degradation”). (4) Health Benefits: (“health benefit*” OR “antioxidant” OR “anti-inflammatory” OR “neuroprotective” OR “cardiovascular” OR “anticancer” OR “antidiabetic” OR “obesity”). (5) Delivery Systems: (“delivery system” OR “encapsulation” OR “microencapsulation” OR “nanoencapsulation” OR “nanoparticle” OR “liposome” OR “emulsion” OR “protein complex” OR “polysaccharide” OR “hydrogel”). The search strings were tailored to fit the specific syntax requirements of each database. Detailed search strategies for all databases can be found in the Supplementary Files.

### 2.2. The Workflow for Study Selection and Screening

**Figure 1:**
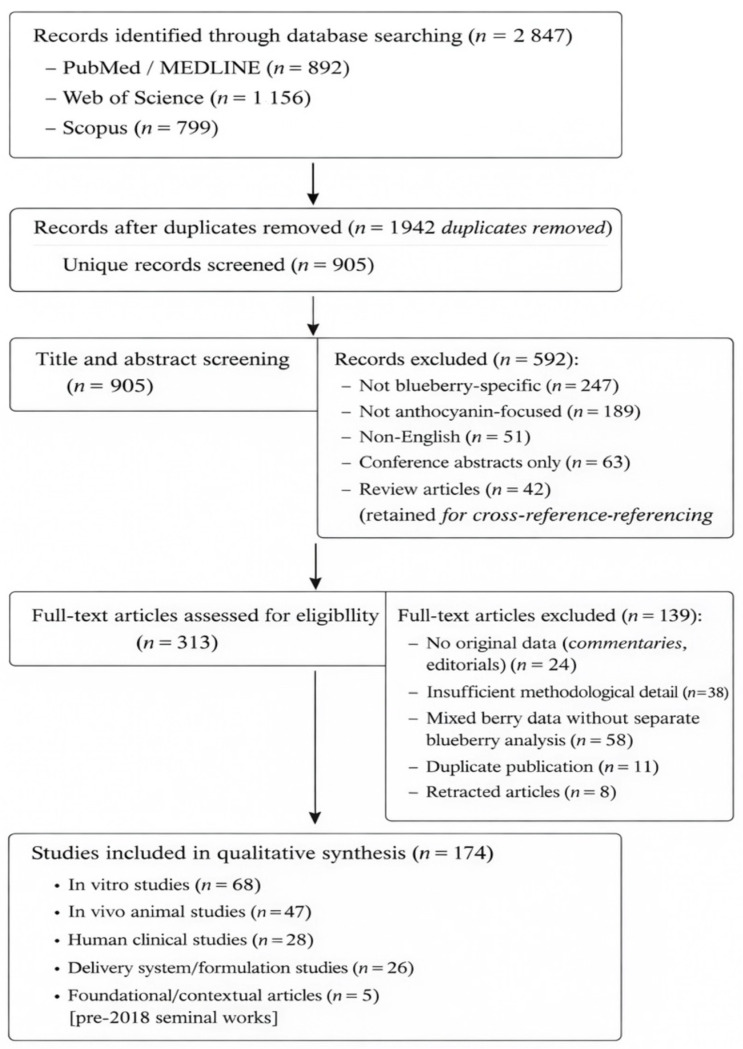
Screening procedure visual representation.

The screening procedure adhered to the PRISMA-ScR guidelines [31] to ensure clarity and reproducibility. Figure 1 provides a visual representation of this workflow.

### 2.3. The Criteria for Inclusion and Exclusion

Studies were considered for inclusion if they satisfied the following criteria: 1. Population: Research focused on blueberry (*Vaccinium* spp.) anthocyanins or blueberries as the main source (over 80%) of anthocyanins studied. 2. Intervention/Exposure: Studies involving the consumption of blueberries, anthocyanin extracts, purified anthocyanins, and anthocyanin-enriched formulations. 3. Comparators: Not applicable for this narrative synthesis; all types of study designs (controlled and uncontrolled) were included. 4. Results: Research reporting on mechanisms of action related to health benefits (in vitro, in vivo, or clinical), factors affecting the stability and bioavailability of anthocyanins, novel formulations, delivery systems, and processing methods to improve bioavailability, and pharmacokinetic parameters (absorption, distribution, metabolism, excretion) 5. Study Design: Original research articles (in vitro, in vivo animal studies, human clinical trials, observational studies) and systematic reviews were included. Conference abstracts, commentaries, editorials, and non-peer-reviewed sources were excluded. 6. Language: English only. 7. Timeframe: 2018–2025, with selective inclusion of key pre-2018 studies for foundational context (e.g., early structural characterization and significant clinical trials).

The studies were excluded based on criteria such as: 1. Research focusing on other types of berries (e.g., strawberry, raspberry, blackberry) without distinct data on blueberries. 2. Investigations involving mixed berry extracts where the specific impact of blueberries could not be determined. 3. Publications not in English were excluded. 4. Conference abstracts that did not provide comprehensive methodological details. 5. Articles that had been retracted.

### 2.4. Thematic Categorization and Extraction of Data

For each study included, the following details were gathered and organized into evidence tables: 1. Study characteristics: This included the authors, publication year, country of origin, study design, and sample size (for studies involving humans). 2. Anthocyanin characterization: Information on the type of blueberry, anthocyanin profile, extraction method, and dose or concentration used. 3. Health benefit outcomes: This covers specific biomarkers, molecular pathways, and clinical endpoints of the study. 4. Delivery system parameters: Includes the type of carrier, particle size, zeta potential, encapsulation efficiency (%), release kinetics, and stability. 5. Gut microbiota interactions: Focuses on microbial metabolites and alterations in microbiota composition. Thematic categorization of the studies included has been provided in the Supplementary File Table S1.

### 2.5. The Synthesis and Analysis of the Data

In light of the narrative and integrative approach of this review, a formal meta-analysis was not performed. Instead, a thematic synthesis strategy was adopted to systematically organize and interpret the literature included in this review. Initially, a synthesis was conducted within each category, where findings from studies within each thematic group were summarized descriptively, emphasizing the consistency of results across studies, the quality and limitations of the methodologies, and the strength of evidence distinguishing preclinical from clinical data. Subsequently, cross-category integration was performed to combine mechanistic insights from in vitro studies with in vivo animal data and human clinical evidence, thereby constructing a comprehensive understanding of anthocyanin bioactivity and bioavailability. For studies that reported on delivery system characteristics, a quantitative parameter synthesis was undertaken, extracting and compiling key data such as particle size, zeta potential, encapsulation efficiency, and release parameters (Table 5) to enable a systematic comparison across various delivery platforms. Furthermore, priority weighting was applied within each theme, giving precedence to the most recent studies (2020–2025), randomized controlled trials, and robust clinical studies for human evidence, as well as investigations with well-defined mechanistic insights and validated methodologies and research presenting novel or significant advancements in delivery technologies. A gap analysis was performed to identify recurring limitations, inconsistent results, and areas lacking sufficient study. These findings were then compiled and elaborated upon in the “Future Challenges” section (Section 9.3) and the conclusion.

### 2.6. Methodology Limitations

It is important to recognize the methodological limitations of this review to accurately interpret the findings. First, the exclusive focus on English-language publications may result in language bias, potentially overlooking relevant studies published in other languages. Second, the timeframe of 2018–2025 was deliberately selected to capture recent advancements in the field; however, this choice might inadvertently exclude older foundational studies. However, key seminal works published before 2018 were selectively included to provide essential context. Third, the narrative and integrative nature of this review precluded the performance of a formal meta-analysis, which limited the ability to estimate quantitative effect sizes and draw statistical conclusions. Fourth, there was considerable heterogeneity among the included studies, particularly regarding study design, anthocyanin sources, dosage regimens, and outcome measures, which restricted direct comparability and complicated the synthesis of findings. Lastly, as with all literature reviews, there is an inherent risk of publication bias, where studies with positive or significant results are more likely to be published and thus over-represented in the available literature, potentially skewing the evidence. These limitations should be considered when interpreting the conclusions of this review.

## 3. Background and Compositional Description of the Blueberries

Blueberries, belonging to the family Ericaceae, subfamily Vacciniaceae, genus *Vaccinium*, and subgenus *Cyanococcus*, are native to North America and are perennial deciduous or evergreen shrubs [30]. They are cultivated in North America (including Canada and the United States), China, Japan, Europe, and Southern Hemisphere countries such as Chile, Argentina, New Zealand, and Australia [29]. Cultivated blueberries are categorized into three types based on plant size and chilling needs: (1) highbush (*Vaccinium corymbosum* L., including southern highbush, northern highbush, and half-high highbush), (2) rabbit eye (*V. ashei* Reade), and (3) lowbush (*V. angustifolium* Ait.) [22,29]. Blueberries contain dietary fiber, vitamins, minerals, hydroxycinnamic acids, pterostilbene, resveratrol, and other bioactive compounds [12,17,32]. They are rich in phenolic compounds, particularly anthocyanins, which enhance their health benefits and flavor [28]. These fruits have high water and sugar contents, mainly glucose and fructose, and contain galactose and rhamnose, which are linked to phenolic compounds. Blueberries are rich in organic acids, such as citric and ascorbic acids, minerals such as phosphorus, potassium, and magnesium, and fiber, especially pectin [32,33]. The main bioactive substances are organic acids, phenolic acids, flavonoids, flavanols and anthocyanins [28].

**Figure 2:**
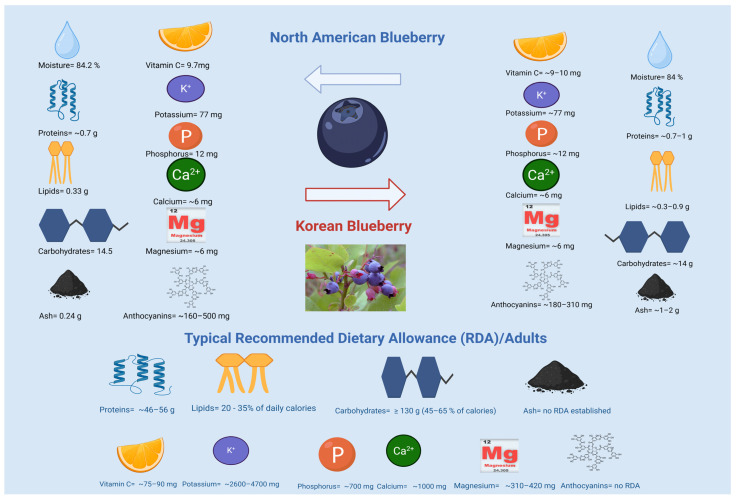
Nutritional composition of North American and Korean blueberries/100 g [13,32]. According to the FDA, the Recommended Dietary Allowance (RDA/adult) is shown in the figure. Images were prepared from https://www.biorender.com/ (accessed on 22 February 2026).

According to the Phenol-Explorer database (http://www.phenol-explorer.eu), the average total phenolic content (TPC) in rabbit eye, highbush, and lowbush blueberries is 5.50, 2.23, and 4.71 mg gallic acid equivalents (GAE)/g fresh weight (FW), respectively. Blueberry fruit comprises 85.78% moisture, 0.73% fat, and 4.10% protein FW basis [32]. Glucose and fructose make up 9.9% of the composition, total anthocyanin content (TAC) is 25–495 mg/g, and chlorogenic acid varies from 0.344 to 1.13 mg/g FW basis [34]. The compositional description of American and Korean blueberries is given in Figure 2. Among phytochemicals, anthocyanins are the most recognized for their health benefits in humans.

## 4. Species and Structure of Anthocyanins from Blueberries

Anthocyanins are pigments that give blueberries their red, purple, and blue colors and play a significant role in the functional attributes of the fruit. The variations in anthocyanin colors are influenced more by the type and position of substituents on the anthocyanidin structure than by glycosylation. Malvidin (Mv) and cyanidin (Cy) glycosides usually exhibit red–purple shades, whereas delphinidin (Dp) glycosides can appear red or blue [35,36]. The color range of blueberry anthocyanins is affected by pH levels, as their ionic nature allows reversible structural changes, resulting in different colors based on acidity or alkalinity [37]. Recent advances in analytical methods, particularly ultra-performance liquid chromatography combined with mass spectrometry (UPLC–MS), have enabled the comprehensive analysis of anthocyanins in blueberries. Wang et al. (2022) [38] examined 62 blueberry varieties and discovered 30 unique anthocyanins from five anthocyanidins, with four glycosides showing three types of structural changes. ‘Rubel’ exhibited the highest total anthocyanin content, with malvidin-galactoside (Malvidin-gal) being the most abundant monomer. Chai et al. [39] investigated 74 blueberry varieties from China and found that delphinidin, malvidin, and petunidin glycosides comprised over 90% of the total anthocyanins, suggesting close genetic ties across breeding lines. The total anthocyanin content in blueberry fruits ranges from 61.8 to 299.6 mg per 100 g of fresh weight [40,41]. Anthocyanins are water-soluble flavonoid pigments with two aromatic rings, labeled A and B, connected by a three-carbon C-ring, creating a C_6_–C_3_–C_6_ framework. Their molecular formula is C_15_H_11_O_6_, and they originate from flavan-3,4-diol precursors, featuring a positively charged c-epoxy atom within the C-ring, which categorizes them as flavonoids [23,29].

The structural variety of anthocyanins results from differences in hydroxylation and methoxylation on the B-ring and glycosylation and acylation, affecting their color, solubility, stability, and bioactivity [29,42]. Glycosylation typically occurs at the C_3_ position of the C-ring, although glycosides may attach to the C_5_ or C_7_ locations on the A-ring, forming mono-, di-, or tri-glycosides. Sugars linked to anthocyanidins include glucose, galactose, rhamnose, arabinose, xylose, and rutinose, which can undergo acylation with aromatic acids such as p-coumaric, caffeic, ferulic, and p-hydroxybenzoic acids or with aliphatic acids such as malonic, acetic, malic, and oxalic acids [23,42,43]. This acylation enhances anthocyanin stability under various pH, temperature, and light conditions, thereby improving their functional properties in food applications [44]. The variety of sugars attached to anthocyanins and the level of acylation determine their solubility, stability, and bioavailability.

**Figure 3:**
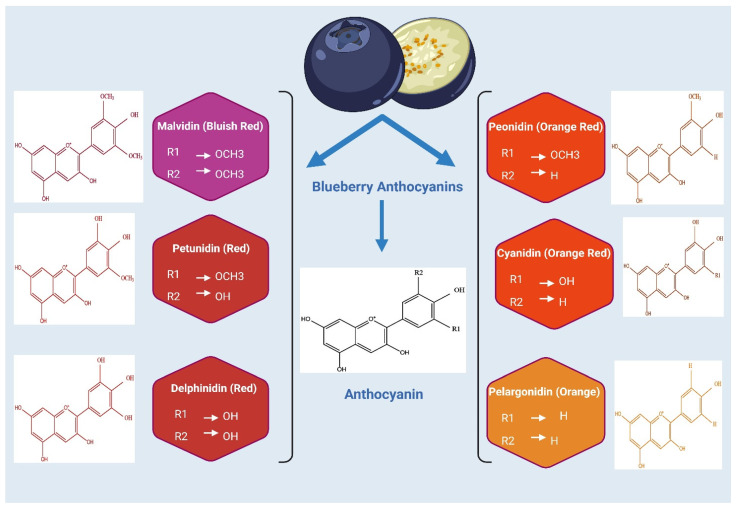
Structure of a typical anthocyanin as well as the six blueberry anthocyanins. Each structure has been elaborated with its typical color and functional groups, i.e., R1 and R2. Images were created using https://www.biorender.com/ (accessed on 22 February 2026) premium software.

Blueberries contain six anthocyanidins: malvidin, delphinidin, petunidin, cyanidin, peonidin, and pelargonidin Figure 3. Malvidin and delphinidin derivatives are usually the most prevalent, followed by petunidin, cyanidin, and peonidin [39,45]. Malvidin content ranges from 21.4% in lowbush varieties (Blomidon and Cignecto) to 43.7% in northern highbush varieties [7,39]. Delphinidin comprises 20.8–56.6% of total anthocyanins and can exceed malvidin levels in certain lowbush and highbush varieties. Petunidin constitutes 7.9–20.6%, cyanidin 4.2–18.2%, and peonidin 0.6–3.8%. These differences are specific to cultivars and are affected by genetic and environmental factors, including soil type, climate, and farming methods [46].

Anthocyanin distribution varies among blueberry varieties. Highbush and lowbush varieties are rich in malvidin and delphinidin, whereas rabbit eye blueberries are rich in peonidin. In Northern and Southern highbush blueberries, petunidin exceeds cyanidin, whereas in some rabbit eye varieties, cyanidin may surpass petunidin [39,47]. This composition influences fruit color and affects antioxidant capacity and health benefits, as different anthocyanidins possess distinct bioactivities.

The remarkable health benefits of blueberries are primarily due to their anthocyanin content, which offers a wide array of biological activities, including potent antioxidant, anti-inflammatory, cardiovascular, neuroprotective, and antidiabetic effects [12,17,30]. The effectiveness of these compounds is closely related to their chemical structures. Key structural elements, such as the level of hydroxylation/methoxylation on the B-ring, glycosylation patterns (such as the type and position of sugar units), and the presence of acyl groups, play crucial roles in determining their chemical stability, absorption rates, and interactions with biological targets [23,29,42]. For example, malvidin glycosides are known for their stability and bioavailability, whereas delphinidin and petunidin derivatives often exhibit superior free radical-scavenging abilities and influence critical signaling pathways, such as NF-κB and MAPK, which regulate inflammation and stress responses [3,48]. Notably, anthocyanins do not function alone; they interact synergistically with other blueberry phytochemicals (such as chlorogenic acids, flavonols, and fibers), which can enhance stability, affect bioavailability, and amplify biological effects, resulting in a combined benefit greater than the sum of the individual parts [28,49]. This intricate phytochemical composition, supported by anthocyanin diversity, forms the basis of the nutritional value of blueberries. Therefore, a detailed understanding of the anthocyanin profiles specific to each cultivar is essential for identifying distinct health benefits, guiding precision breeding for increased bioactive content, and informing the strategic development of functional foods derived from these fruits. Advancements in analytical methods, such as UPLC-MS and metabolomics, are crucial for unraveling these complex structure-activity relationships and tracing the metabolic pathways of anthocyanins, thereby connecting blueberry composition with tangible health outcomes [29,38,50].

## 5. Mechanism of Action of Blueberry Anthocyanins to Incur Health Benefits

### 5.1. Protection to Neural Systems

Neurons are vulnerable to oxidative stress, inflammation, and neurotoxic substances, which contribute to neurological and neuropsychiatric disorders. Major depressive disorder (MDD) is one of the most widespread conditions worldwide, characterized by complex biological mechanisms and limited therapeutic efficacy. Recent findings suggest that blueberries, especially their polyphenol- and anthocyanin-rich components, exhibit significant neuroprotective effects [51]. Studies have indicated that blueberry anthocyanins act as natural neuroprotectants capable of mitigating neuronal damage and enhancing cognitive function. Wang et al. [52] found that anthocyanins from blueberries shielded PC12 cells from hydrogen peroxide-induced oxidative cytotoxicity, reduced lipid peroxidation, and inhibited acetylcholinesterase activity, leading to improved memory outcomes in mice subjected to trimethyltin-induced neurotoxicity. In preclinical models of ketamine-induced mania, blueberry ethanol extract alleviated behavioral impairments and biochemical abnormalities, suggesting its therapeutic potential against mood-related neurotoxicity [53].

Human studies have shown that older adults consuming wild blueberry juice for 12 weeks experienced significant improvements in cognitive performance, indicating that blueberry polyphenols may help combat age-related cognitive decline [54]. Blueberry anthocyanins provide neuroprotection through their antioxidant properties and ability to influence cellular stress pathways. Malvidin significantly decreased reactive oxygen species (ROS) production in Aβ-treated Neo-2a cells, thereby reducing oxidative damage [55]. Blueberry polyphenols boost autophagy and alleviate cellular stress, as shown in a metabolic model where they facilitate autophagy-driven lipid turnover [56], which is important for neuronal resilience. Blueberry anthocyanins protect neural cells from environmental toxins by preventing changes in neural structure and DNA damage caused by perfluoro octane sulfonate exposure [57]. These studies demonstrate that blueberry anthocyanins alleviate oxidative stress, maintain neuronal integrity and function, and promote cognitive health, highlighting their potential as neuroprotective agents.

Persistent inflammation plays a crucial role in the onset of chronic illnesses such as diabetes, cardiovascular disease, arthritis, and metabolic disorders [58]. Blueberries and their extracts, which are rich in anthocyanins, modulate inflammatory processes, offering preventive and therapeutic advantages [59]. In cell models, blueberry anthocyanin extracts reduced inflammation triggered by lipopolysaccharides in RAW 264.7 macrophages by decreasing the expression of inflammatory mediators, such as COX-2, iNOS, and IL-1β, with malvidin-3-β-glucoside identified as the most effective anti-inflammatory agent [48]. Animal studies have shown convincing results for this. In a rat arthritis model, blueberry extract with 0.58 mg/mL anthocyanins mitigated clinical symptoms, such as bone resorption, soft tissue swelling, and osteophyte formation, thereby improving joint mobility and function [60]. In addition, cyanidin-3-glucoside combined with blueberry pectin demonstrated strong protective effects against dextran sodium sulfate-induced ulcerative colitis in mice [59]. Evidence from population studies highlights the anti-inflammatory properties of anthocyanins. In a survey of 2375 individuals, higher consumption of anthocyanin-rich foods, such as blueberries, was linked to lower levels of 12 systemic inflammatory biomarkers [61].

In vitro and animal studies indicate that blueberry anthocyanins may modulate inflammatory signaling pathways. In cellular models, such as RAW 264.7 macrophages, these compounds, often at concentrations exceeding physiological levels (>10 µM), have been found to inhibit the phosphorylation of NF-κB p65 and IκB, as well as suppress the MAPK p38 and JNK pathways, resulting in decreased production of TNF-α and IL-6 [48,62]. Nonetheless, it remains to be definitively proven whether these specific pathway inhibitions occur in human tissues at nanomolar to low micromolar concentrations of anthocyanin metabolites achievable through diet. The anti-inflammatory effects observed in humans are likely due to a combination of indirect mechanisms, including modulation of the gut microbiota and the actions of more prevalent phenolic acid metabolites [48,62]. These results align with those of Roth et al. [63], who found that anthocyanins from European blueberries diminished IFN-γ-induced signal activation and cytokine secretion in THP-1 monocytes. Human intervention studies have shown that blueberry consumption can improve postprandial glucose control and enhance insulin sensitivity by reducing oxidative stress and inflammation in the gastrointestinal tract [64]. In summary, blueberry anthocyanins exhibit potent anti-inflammatory effects in cellular, animal, and human inflammation models. Their ability to modulate inflammatory gene expression and block inflammatory signaling pathways highlights their potential as dietary agents for the management of diseases associated with chronic inflammation.

### 5.2. Immunity Against Cancer

The global incidence of cancer is increasing, with projections indicating that new cases could reach 22 million by 2030 [65]. This trend has spurred research into preventive measures, particularly the use of dietary bioactive compounds. Blueberries, known for their high anthocyanin and polyphenol content, have garnered interest for their potential chemopreventive and anticancer properties, primarily based on promising preclinical evidence [66]. In vitro, in vivo, and clinical studies have shown that blueberry anthocyanins inhibit cancer cell proliferation, trigger apoptosis, influence immune responses, and reduce metastasis. In hepatocellular carcinoma cells (HepG-2), blueberry anthocyanins induced apoptosis by increasing ROS production, disrupting mitochondrial membrane potential, activating caspase-3, and promoting cytochrome c release from mitochondria. These processes are associated with the downregulation of the anti-apoptotic protein Bcl-2 and upregulation of the pro-apoptotic factor Bax, indicating the activation of the intrinsic apoptotic pathway [30,67,68]. It is crucial to recognize that the concentrations required to achieve these effects, often ranging from 10 to 100 µM, generally exceed the nanomolar to low micromolar levels of anthocyanin metabolites attainable in human plasma through dietary consumption of anthocyanin-rich foods [69,70].

Comparable antiproliferative effects have been observed in breast cancer models. Faria et al. [71] found that blueberry anthocyanin extracts (250 μg/mL) and anthocyanin-pyruvate adduct inhibited the growth of MDA-MB-231 and MCF-7 breast cancer cells. In colon cancer HT-29 cells, blueberry extracts induced apoptosis, partly by inhibiting COX-2 signaling, a pathway that is often upregulated in colorectal tumors [7]. Animal studies have corroborated these anticancer benefits in humans. In CD-1 mice with tumors, blueberry polyphenol extracts boosted the immune response and slowed tumor growth. Nonetheless, the dosages used in rodent studies cannot be directly converted to equivalent human doses, and the tumor models may not accurately reflect the diversity of human cancers [72].

Mechanistic studies have revealed that blueberry anthocyanins influence several pathways crucial for cancer development. Cyanidin-3-rutinoside triggers ROS-dependent programmed cell death in HL-60 leukemia cells by activating p38 MAPK and JNK, thereby initiating the mitochondrial apoptotic pathway [73]. In vitro studies have demonstrated that blueberry extracts and isolated anthocyanins, often used in high concentrations ranging from tens to hundreds of µM, can suppress proliferation and pathways related to metastasis in breast cancer cell lines. These effects are linked to the modulation of the PI3K/Akt, MAPK/ERK, and STAT3 pathways [74]. A major challenge in translating these findings is that the concentrations used in these studies are much higher than the peak plasma levels of the parent compounds (<100 nM) observed in humans after consuming blueberries [69,70]. This difference necessitates caution when applying these specific molecular mechanisms to dietary cancer prevention. Purified anthocyanins, such as cyanidin and its glycosides, reduce the proliferation of A549 lung cancer cells at concentrations ranging from 100 to 300 μM, demonstrating their anticancer potential [75]. In humans, chemopreventive potential may depend more on the combined effects of various blueberry components and their metabolites at lower concentrations [74].

Blueberries play a crucial role in modulating the immune system, which is a key aspect of their anticancer properties. Immune response imbalance is a significant factor in tumor onset and progression. Malvidin, found in blueberry extracts, decreases inflammatory markers MCP-1, ICAM-1, and VCAM-1 in endothelial cells by affecting IκBα degradation and preventing nuclear movement of NF-κB p65, thereby reducing inflammation-related cancer signaling [76,77]. These studies underscore the diverse anticancer capabilities of blueberry anthocyanins, including the promotion of apoptosis, curbing of cell proliferation, limiting of metastasis, and regulation of the immune system.

There is still a scarcity of direct clinical evidence supporting the anticancer effects in humans, with most data being epidemiological. Although diets high in anthocyanins are linked to a lower risk of cancer in population studies [17], there is a lack of strong interventional trials showing tumor reduction effects. This underscores the need for additional research to determine whether the promising mechanistic actions observed in preclinical studies can be translated into therapeutic or preventive benefits for humans.

### 5.3. Protective Effects on Eyes and Vision

Evidence indicates that anthocyanins in blueberries have protective effects on visual function, particularly during oxidative stress and retinal damage. Research has shown that blueberry anthocyanin extracts and compounds such as malvidin, malvidin-3-glucoside, and malvidin-3-galactoside reduce oxidative damage from hydrogen peroxide (H_2_O_2_) in human retinal pigment epithelial (RPE) cells [78]. These protective effects stem from the antioxidant properties of anthocyanins and their ability to maintain retinal cell integrity. In diabetic models, blueberry anthocyanins protect retinal cells from oxidative stress and inflammation. Song et al. [79] found that anthocyanin supplementation reduced retinal damage in diabetic rats by activating the Nrf2/HO-1 signaling pathway, crucial for cellular antioxidant defense. Another study showed that anthocyanins, including pelargonidin-3-glucoside, cyanidin-3-glucoside, delphinidin-3-glucoside, and malvidin-3-glucoside, from berries protected human RPE cells from visible light damage. Cyanidin-3-glucoside, with its ortho-hydroxyl group on the B-ring, exhibits strong antioxidant, anti-angiogenic, and anti-aging properties, suggesting its potential as a preventive nutraceutical for retinal disorders [80].

Limited human intervention studies have provided insights into this area. A randomized controlled study assessed the impact of blueberry anthocyanins on visual performance in low-light conditions, night vision, and dark adaptation after retinal photobleaching. Two trials used different dosages and durations. Trial 1 involved 3-week treatment and washout with doses of 271 and 7.11 mg cyanidin-3-glucoside (*n* = 72), while Trial 2 examined longer supplementation with 346 mg cyanidin-3-glucoside equivalent over 8 and 12 weeks (*n* = 59). Although neither trial improved night vision or dark adaptation, blueberry anthocyanin intake improved visual function recovery after photobleaching at both dosage levels and durations. The clinical significance of faster photobleaching recovery for daily visual performance requires further investigation [81].

### 5.4. Protection Against Cardiovascular Diseases

Cardiovascular diseases (CVDs) are the leading cause of death worldwide, particularly in industrialized countries. Research indicates that diet influences CVD risk, with anthocyanin-rich fruits, such as blueberries, linked to heart health benefits [12]. Blueberry anthocyanins provide vascular protective effects, such as altering lipid metabolism, improving endothelial function, reducing inflammation, and decreasing oxidative stress. In an ApoE^−^/^−^ (Apolipoprotein E knockout mouse) model of atherosclerosis, the consumption of freeze-dried blueberry powder over 20 weeks led to a 39% decrease in aortic sinus lesion area and a 58% reduction in descending aortic lesions, highlighting its anti-atherogenic properties [82]. Human studies have also supported these findings. In people with high cholesterol, daily intake of purified blueberry anthocyanins, specifically delphinidin-3-O-β-glucoside and cyanidin-3-O-β-glucoside, reduced inflammatory markers, such as serum hs-CRP, sVCAM-1, and plasma IL-1β. These compounds also lower LDL cholesterol and raise HDL cholesterol levels, thereby improving the lipid profiles [12]

Blueberry anthocyanins offer cardiovascular benefits by impacting molecular pathways. Their antioxidant capabilities help mitigate ROS-induced vascular damage, a major cause of endothelial dysfunction and atherogenesis [83]. These anthocyanins influence critical signaling pathways that maintain vascular balance, such as NF-κB, MAPK, and Nrf2 pathways, which decrease inflammation, boost antioxidant defenses, and protect endothelial cells. Anthocyanins affect the expression of regulatory microRNAs (miRNAs). In ApoE^−^/^−^ mice, purified anthocyanins modified the expression of 54 hepatic miRNAs associated with lipid metabolism and vascular inflammation. Research has shown that these miRNA-driven changes contribute to reduced endothelial cell permeability and enhanced endothelial function [84]

Epidemiological research supports a link between anthocyanin consumption and cardiovascular health benefits. Regular consumption of foods high in anthocyanins, particularly blueberries, is associated with a reduced risk of death from cardiovascular disease [12]. The anthocyanins in blueberries provide heart-protective benefits through antioxidant properties, anti-inflammatory pathways, better lipid metabolism, improved endothelial function, and modulation of miRNA expression, making them effective natural options for the prevention and management of cardiovascular diseases.

### 5.5. Mitigating and Inhibitive Effects Against Type 2 Diabetes

Type 2 diabetes mellitus (T2DM) accounts for over 90% of diabetes cases globally and is characterized by insulin resistance, beta-cell failure, and high blood sugar levels. Lifestyle and dietary habits, such as excessive consumption of high-fat foods, ultra-processed products, and a lack of physical activity, are crucial in the increasing incidence of T2DM [85]. Growing evidence indicates that the daily consumption of blueberries and their anthocyanin-rich extracts may offer an effective method for preventing and managing T2DM through various mechanisms [12]. In experimental studies, obese Zucker rats fed blueberry powder showed decreased plasma HbA1c, retinol-binding protein 4 (RBP4), and resistance levels, suggesting improved blood sugar control [86]. Male KK-Ay mice administered European blueberry extract exhibited activation of AMP-activated protein kinase (AMPK), which enhanced glucose transporter 4 (GLUT4) movement, increased glucose uptake, reduced liver glucose production, and lowered blood glucose levels while improving insulin sensitivity [87].

Clinical trials have shown that the consumption of 22 g of freeze-dried blueberries daily for eight weeks positively impacts cardiometabolic parameters in men with T2DM, including improvements in vascular function and inflammation [88]. Blueberry anthocyanins reduce carbohydrate digestion by inhibiting intestinal α-amylase and α-glucosidase activities, thereby lowering post-meal glucose spikes. These anthocyanins enhance glucose uptake by activating PPAR-α and PPAR-γ in muscle and fat tissues, boosting insulin secretion, and improving insulin receptor signaling [17]. They influence the gut microbiota by increasing beneficial Bifidobacteria, which are linked to better insulin receptor expression and improved glucose homeostasis [89]. The anti-inflammatory and antioxidant properties of these compounds help mitigate oxidative stress-induced beta cell dysfunction and inflammation, which are significant factors in insulin resistance and T2DM progression. Through these mechanisms, blueberry anthocyanins provide a comprehensive approach to T2DM management.

### 5.6. Impact on Metabolic Dysfunction Linked to Obesity

Obesity significantly increases the risk of metabolic disorders, such as T2DM, cardiovascular diseases, and systemic inflammation [12,88]. Dietary strategies that address the root causes of metabolic imbalances are essential. Studies on blueberries and their anthocyanins suggest that their main advantage may be alleviating the metabolic issues associated with obesity rather than consistently promoting weight loss. Evidence from preclinical models regarding the effects on body fat varies. Some studies, especially those utilizing concentrated forms such as purified anthocyanins or freeze-dried powder, indicate reduced weight gain and fat accumulation in diet-induced obese rodents [89], while other studies show little effect on weight. For example, wild blueberry powder did not significantly change body weight in certain models but consistently enhanced important metabolic parameters such as glucose regulation and insulin sensitivity [90]. Interestingly, some formulations have been linked to weight gain in specific mouse models, yet still offer metabolic benefits, such as improved glucose tolerance and decreased liver fat [90]. This separation highlights the fact that the metabolic advantages of blueberry anthocyanins can occur without changes in body weight. Human clinical trials have generally reflected this trend. Interventions such as the daily intake of freeze-dried blueberry powder often do not lead to significant weight loss but consistently improve metabolic syndrome biomarkers, including vascular function and inflammatory markers [7,86]. A promising approach seems to be replacing other carbohydrate sources with blueberries in an isocaloric manner, which has shown reductions in body weight and fat, indicating that their role in a balanced diet may be more effective for weight management than supplementation alone [7]. The mechanisms by which blueberry anthocyanins enhance metabolic health are well documented. They influence adipocyte function and systemic energy regulation. For instance, cyanidin can increase the expression of genes involved in lipid metabolism, boost hormone-sensitive lipase activity, and promote the secretion of beneficial adipokines such as adiponectin [91]. Similarly, malvidin glycosides have been found to prevent lipid buildup in adipocytes exposed to free fatty acids [92]. These actions collectively encourage lipolysis, enhance insulin sensitivity, and restore glucose and lipid balances. In conclusion, although blueberry anthocyanins are not guaranteed weight-loss agents, they have a strong ability to address key metabolic disturbances such as insulin resistance, dyslipidemia, and inflammation, which are characteristic of obesity. Therefore, their greatest value may lie in being a dietary component that improves metabolic health and reduces the risk of obesity-related diseases, regardless of absolute weight change [92,93].

### 5.7. Antioxidant Properties

Anthocyanins are strong antioxidants owing to their chemical composition, which enables them to donate hydrogen atoms from the phenolic ring to counteract free radicals. Their antioxidant strength is approximately 50 and 20 times that of vitamin E and vitamin C, respectively, underscoring their potential as natural antioxidants [94]. Blueberries, which are abundant in anthocyanins and anthocyanidins, are recognized for their antioxidant properties, as documented in laboratory and live studies [29]. Anthocyanins and anthocyanidins contribute to the overall antioxidant capacity of blueberry phytochemicals, accounting for 84% of the total antioxidant potential. Human studies have shown that the consumption of 100 g of freeze-dried blueberry powder, containing 1.2 g of anthocyanins (42% of total phenolic content), significantly increased plasma antioxidant capacity, suggesting that dietary anthocyanins enhance systemic oxidative defense [30].

Research involving animals and cell cultures has reinforced these conclusions [30,45]. ApoE^−^/^−^ (Apolipoprotein E knockout mice) rats lacking vitamin E and given blueberry anthocyanins experienced significant decreases in lipid peroxidation, whereas endothelial cells under oxidative stress showed enhanced protection when treated with these anthocyanins [4]. The antioxidant benefits extend beyond radical scavenging ability. These compounds influence the body’s antioxidant systems and reduce oxidative enzyme activity, thereby prolonging cellular defense.

**Figure 4:**
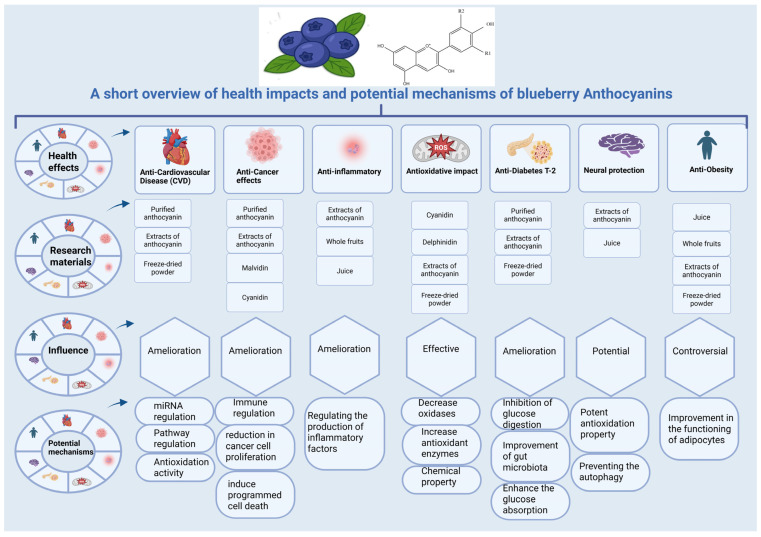
The figure illustrates a short overview of health effects, research materials, influence, and potential mechanisms of blueberry anthocyanins. The illustration was prepared using https://www.biorender.com/ (accessed on 22 February 2026).

According to Yang et al. [29], Blueberry and cranberry juices, rich in cyanidin derivatives, enhance mitochondrial activity and reduce oxidative stress in neuroblastoma SH-SY5Y cells exposed to hydrogen peroxide. They decrease reactive oxygen species and lipid peroxidation while upregulating antioxidant enzymes catalase and superoxide dismutase. ORAC and FRAP assays confirmed their antioxidant capacity, supporting their potential use in pharmaceutical and nutritional approaches to prevent neuronal oxidative injuries. In addition to directly scavenging radicals, anthocyanins can also trigger the body’s own antioxidant defenses. Preclinical research has demonstrated that compounds such as cyanidin, when used at experimental levels, can activate the Nrf2 transcription factor. This activation results in increased production of protective enzymes, such as HO-1 and NQO1, in endothelial cells under stress [95]. Activation of the Nrf2 pathway is a potential mechanism that may function at lower, physiologically relevant metabolite concentrations. However, direct evidence showing that blueberry-derived metabolites modulate the Nrf2 pathway in human tissues at levels typical of dietary exposure is lacking. These findings indicate that blueberry anthocyanins directly neutralize free radicals and activate internal defense mechanisms, thereby offering a comprehensive approach to mitigate oxidative stress. These characteristics highlight the potential of blueberry anthocyanins as natural antioxidants with therapeutic significance in preventing diseases related to oxidative stress, including cardiovascular disease, diabetes, neurodegeneration, and cancer. The well-documented antioxidant potential of blueberry anthocyanins is evident in both chemical (ORAC and FRAP) and cellular evaluations; however, their role as direct antioxidants in humans remains complex and debated. Owing to the low systemic bioavailability of these compounds, their direct radical scavenging activity in tissues is likely to be minimal. Instead, their health benefits may be indirectly facilitated by (1) the antioxidant and anti-inflammatory properties of their more prevalent phase II and microbial metabolites, (2) the enhancement of the body’s endogenous antioxidant defenses, such as through Nrf2 activation, and (3) localized effects within the gastrointestinal tract [95,96]. Therefore, although blueberries are abundant in dietary antioxidants, their health benefits are not likely to be attributed to simple antioxidant action. Rather, these benefits are likely the result of a complex interaction involving subtle and diverse adjustments to redox and inflammatory signaling pathways. Multiple and potential health impacts and mechanisms of action are shown in Figure 4. Similarly, blueberry anthocyanin health benefits with in vitro, animal, and human evidence, as well as concentration gaps and physiological plausibility across health domains, are presented in Table 2 and Table 3, respectively.

## 6. Mechanistic Overview of Bioavailability and Metabolism of Blueberry Anthocyanins

The bioavailability of blueberry anthocyanins, which refers to the fraction of the consumed dose that enters the systemic circulation in an active form, is crucial for their health benefits. A contemporary perspective goes beyond mere absorption to encompass the entire Absorption, Distribution, Metabolism, and Excretion (ADME) profile, which is significantly influenced by both host and microbial biotransformation [99,100].

### 6.1. Gastrointestinal Absorption and First-Pass Metabolism

After consumption, a minor portion (<2%) of intact anthocyanin glycosides is absorbed in the stomach and small intestine through passive diffusion and facilitated transport mechanisms (e.g., via SGLT1 and GLUT2) [23,101]. This is followed by extensive phase II conjugation in enterocytes and hepatocytes, during which anthocyanins undergo glucuronidation, methylation (via catechol-O-methyltransferase), and sulfation [28,97]. These reactions, illustrated in Figure 4, produce various conjugated metabolites (e.g., cyanidin-3-glucoside-glucuronide and peonidin-3-glucoside-sulfate), which are often more stable and biologically active than the original compounds and represent the main circulating forms [97,102].

### 6.2. Microbial Metabolism and Key Catabolites

More than 95% of anthocyanins consumed are not absorbed in the colon, where they serve as substrates for gut bacteria. Enzymes produced by these bacteria, such as β-glucosidases, reductases, demethylases, and decarboxylases, break down glycosidic bonds and split the anthocyanin C-ring, yielding various low-molecular-weight phenolic catabolites. Important microbial metabolites include protocatechuic acid (derived from cyanidin), vanillic acid (derived from peonidin), syringic acid (derived from malvidin), and phthalide derivatives [98,103]. These catabolites, which can enter the bloodstream, often have longer half-lives and exhibit notable anti-inflammatory and antioxidant properties, significantly contributing to the systemic benefits of blueberry consumption [24,104].

### 6.3. Realistic Biomarkers and Analytical Insights

**Figure 5:**
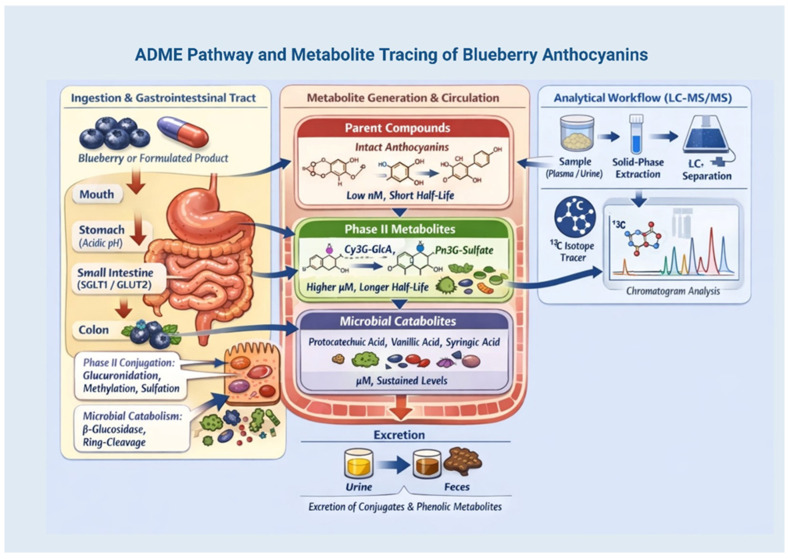
The diagram shows the Absorption, Distribution, Metabolism, and Excretion (ADME) processes of blueberry anthocyanins after consumption. It shows limited absorption of intact glycosides in the upper gastrointestinal tract, Phase II conjugation (including glucuronidation, methylation, and sulfation) in enterocytes and the liver, and microbial breakdown in the colon, producing phenolic acid metabolites such as protocatechuic acid. Metabolite profiles in blood and urine are analyzed by liquid chromatography–tandem mass spectrometry (LC-MS/MS), often with stable-isotope-labeled tracers for pharmacokinetic analyses.

Owing to their rapid metabolism, intact anthocyanins are found in plasma at only nanomolar concentrations, with peak levels (Cmax) occurring 1–2 h after consumption [28,105]. In contrast, their phase II conjugates and microbial phenolic acids reach higher concentrations (micromolar) and remain longer, making them more reliable biomarkers of intake and exposure in clinical research [97,105]. Advanced analytical techniques, especially liquid chromatography coupled with tandem mass spectrometry (LC-MS/MS) and the use of stable isotope-labeled tracers (e.g., ^13^C-cyanidin-3-glucoside), have been crucial in unraveling these intricate metabolic pathways and measuring trace levels of metabolites in biological fluids (Figure 5) [97,102].

### 6.4. Influence of Food Matrix and Formulation

The composition of the food matrix and delivery method play crucial roles in influencing ADME. Whole blueberries, which contain natural fiber and co-pigments, can slow gastric emptying and create a protective environment, resulting in a more prolonged release and elevated levels of microbial metabolites compared to juices or purees [70,106]. Conversely, engineered delivery systems, such as protein complexes, nanoliposomes, and polysaccharide-based carriers, are designed to enhance stability in the upper gastrointestinal tract. Notably, their impact goes beyond boosting the absorption of the original compound; they significantly alter the systemic metabolite profile, affecting both the kinetics and relative abundance of phase II conjugates and microbial catabolites [26,107,108]. Understanding these formulation-metabolite interactions is essential, as the health benefits of anthocyanins are increasingly linked to their metabolites rather than the original compounds [97,105]. Understanding these matrix effects is vital for developing functional foods with predictable pharmacokinetics (PK). Some evidence-based examples of protein complexes, polysaccharides, lipids, and composite carriers are given below to illustrate the influence of formulations on metabolite profiles.

Lang et al. [106] found that when blueberry anthocyanins form a complex with α-casein, they not only enhance their stability in the stomach but also significantly change the urinary metabolite profile in rats. In comparison to free anthocyanins, the α-casein complex led to a 22–35% increase in the relative abundance of methylated and glucuronidated conjugates, such as peonidin-3-O-glucoside-glucuronide, while decreasing the excretion of sulfated derivatives. This suggests that protein complexation may influence the activity of intestinal catechol-O-methyltransferase (COMT) and UDP-glucuronosyltransferases (UGTs), promoting specific flavonoid conjugation pathways. Similarly, whey protein complexes have been shown to postpone the release of anthocyanins in the small intestine, resulting in a prolonged Tmax and elevated Cmax for phase II metabolites compared to free anthocyanins [109,110].

Colonic delivery systems, such as chitosan-pectin nanoparticles and alginate microcapsules, have been shown to enhance the production of phenolic acids derived from the gut microbiota. According to Flores et al. [107], encapsulating anthocyanins in cyclodextrin complexes resulted in a 2.5-fold increase in the production of protocatechuic acid (from cyanidin) and syringic acid (from malvidin) during in vitro fermentation with human fecal microbiota compared to anthocyanins that were not encapsulated. This increase was attributed to the controlled release of intact anthocyanins into the colon, where they acted as substrates for bacterial β-glucosidases and ring cleavage enzymes. In vivo studies have linked chitosan-based nanoparticles to elevated fecal levels of phenyl propionic and phenylacetic acids, metabolites associated with enhanced gut barrier function and anti-inflammatory effects [98,108].

Nanoliposomes and solid lipid nanoparticles (SLNs) mainly safeguard anthocyanins in the upper gastrointestinal tract, resulting in improved absorption of intact glycosides. According to Chi et al. [111], anthocyanins encapsulated in nanoliposomes led to a higher percentage of intact anthocyanins in plasma (increased by 18–25%) than phase II conjugates, as opposed to free anthocyanins. This alteration in the ratio of parent compounds to metabolites could affect bioactivity, as intact glycosides and their conjugated metabolites engage with different molecular targets within the body. Nonetheless, SLNs and nanostructured lipid carriers (NLCs) have been noted to encourage enterohepatic recirculation, causing a delayed appearance and extended circulation of glucuronidated and sulfated metabolites [112,113].

Innovative composite systems, such as chitosan-coated liposomes and protein-polysaccharide coacervates, have been engineered to offer gastric protection, regulate release in the small intestine, and ensure precise delivery to the colon. Wang et al. [114] illustrated that a dual-layer nanocomplex made of chitosan hydrochloride, carboxymethyl chitosan, and whey protein isolate exhibited a triphasic release pattern: less than 10% release in simulated gastric fluid, a gradual 40–50% release in simulated intestinal fluid, and a prolonged release of remaining anthocyanins in the colon. This led to a biphasic plasma metabolite profile in rats, with an initial peak of phase II conjugates (from absorption in the small intestine) occurring at 1–2 h, followed by a later peak of phenolic acid metabolites (from colonic fermentation) at 6–8 h. Such targeted delivery across multiple compartments could enhance both systemic and gut health. The following Table 4 shows the evidence-weighted examples of formulation effects on anthocyanin metabolite profiles.

These examples, weighted by evidence, highlight that the selection of a delivery system influences not only the level of anthocyanin absorption but also the qualitative and quantitative profiles of metabolites in circulation and excretion in urine. Consequently, future formulation development should prioritize metabolite profiling as a crucial outcome measure rather than concentrating solely on the bioavailability of the parent compound. Table 2 summarizes the relationships between the formulations and metabolites.

## 7. In Vitro, In Vivo, and Human Studies

### 7.1. In Vitro Research

Anthocyanins have limited bioaccessibility; however, blueberries are a better source than raspberries, blackberries, and strawberries [12,29]. In vitro gastrointestinal models incorporating cellulose dialysis membranes or Caco-2 cell monolayers mimic minimal intestinal drug absorption. These models help assess the proportion of anthocyanins absorbed into the system (“IN” fraction) versus the portion reaching the colon for microbial breakdown (“OUT” fraction). In blueberry digestion studies, 0.05 mg/g of anthocyanins were found in the IN fraction, while 0.21 mg/g were found in the OUT fraction. Although the fractional bioaccessibility is lower, the higher anthocyanin content in blueberries ensures significant delivery compared to other fruits [49]. The bioaccessibility of anthocyanins depends on their chemical structure, glycosylation pattern, presence of methoxyl or hydroxyl groups on the B-ring, and food matrix, including soluble and insoluble fibers. Anthocyanins with fewer hydroxyl groups or methoxylated aglycones are more stable in the gastric and intestinal environments. Specific glycosylation patterns, such as those of glucosides and galactosides, can enhance absorption through intestinal epithelial cells. Interactions with digestive enzymes, such as α-amylase, may decrease flavonoid glycoside bioaccessibility, affecting absorption [122].

Research using Caco-2 monolayers has shown that anthocyanin transport depends on structure, with hydrophobic anthocyanins showing higher transcellular transport. Food matrices significantly affect the absorption of nutrients. Anthocyanins in complex matrices, such as whole blueberries, may be released more slowly with lower solubility. Processed versions, such as juices, could enable quicker absorption due to the absence of cell walls and fibers [101]. While in vitro models with artificial barriers and cell monolayers provide insights into potential absorption, they cannot fully replicate in vivo digestion, which involves enzyme interactions, changes in pH, and microbial metabolism.

### 7.2. In Vivo Research

In vivo studies have shown that anthocyanins have low absolute systemic bioavailability, generally between 0.26% and 1.8% of the doses consumed [100]. Despite their low plasma concentrations, anthocyanins and their metabolites have been detected in various tissues, indicating potential systemic effects. In female athymic mice fed a diet containing 5% blueberries, delphinidin and cyanidin were present in lung tissue only after conversion to aglycones through hydrolysis, suggesting that anthocyanins can reach peripheral tissues and influence local biological activities [123]. In ovariectomized Sprague-Dawley rats, cyanidins and malvidins were primarily excreted in the urine as unmetabolized substances, whereas delphinidins and peonidins underwent glucuronidation and sulfation [100]. This variation underscores the impact of structural characteristics on the stability and systemic persistence of anthocyanins. Anthocyanin bioavailability is complicated by its kinetics. These compounds have short half-lives in the bloodstream, leading to low detection rates at specific sampling intervals.

Research on isotopically labeled anthocyanins in rodents has shown their native and metabolized forms in blood and urine, suggesting that rapid metabolism does not prevent systemic circulation or biological activity [50]. Additionally, di-glycosylated anthocyanins, such as cyanidin and malvidin derivatives, resist pH-induced degradation during stomach and intestinal passage, enabling more ingested doses to withstand digestion and reach the colon.

**Figure 6:**
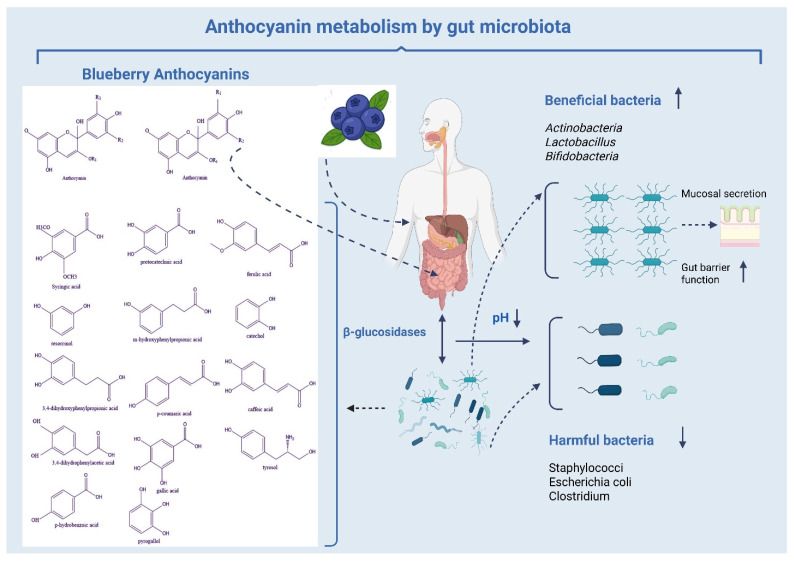
The figure illustrates the mechanism of blueberry anthocyanins in gut microbiota and resulting metabolites using enzymes and pH. The figure was created using https://www.biorender.com/ (accessed on 22 February 2026).

The gut microbiota significantly influences the bioavailability and effectiveness of anthocyanins. The metabolism of blueberry anthocyanins is shown in Figure 6. Bacteria in the colon break down anthocyanin glycosides into phenolic acids and smaller metabolites with extended half-lives that can enter the bloodstream, enhancing systemic antioxidant and anti-inflammatory properties.

Microbiota-mediated metabolism generates compounds that affect gut barrier integrity, glucose metabolism, and immune signaling, linking anthocyanin consumption to broader health benefits. This interaction between anthocyanins and the gut microbiota emphasizes the importance of considering both direct absorption and microbial transformation when evaluating bioavailability. The method of anthocyanin consumption affects their bioavailability. Research comparing blueberry juice to smoothies shows that juice may lead to better systemic availability, likely because juice has fewer interactions with fibers and moves more quickly through digestion [50,123,124]. Regular consumption of anthocyanins may enhance their accumulation in tissues or metabolites, sustaining physiological effects despite low plasma levels. These findings show that bioavailability depends on digestion, metabolism, microbial transformation, and consumption patterns.

Laboratory and animal studies have indicated that blueberry anthocyanins can enter the bloodstream in their active forms, interact with tissues, and exert biological effects, despite low absorption rates. Factors such as structural properties, glycosylation patterns, food matrix, microbial metabolism, and consumption influence their bioavailability. Understanding these elements is crucial for comprehending systemic effects and enhancing dietary strategies. Additional research, particularly human clinical trials, is needed to improve pharmacokinetic models and identify the optimal dose and form of blueberries for health benefits.

### 7.3. Studies About the Bioavailability in Humans

Research on blueberry anthocyanin bioavailability in humans has yielded findings regarding their absorption, metabolism, and presence in the bloodstream. After blueberry consumption, the peak plasma levels of total anthocyanins vary between 1 and 100 nmol/L, indicating limited absorption and swift metabolism [28]. These studies were conducted under strict ethical guidelines, requiring informed consent from the participants and institutional ethics board approval. Participants were healthy adults aged 20–45 years without cardiovascular, metabolic, renal, hepatic, gastrointestinal, or respiratory issues and were not on medications affecting anthocyanin metabolism. Studies accounted for body mass index and smoking habits to minimize confounding factors, ensuring that pharmacokinetic and metabolic results were linked to blueberry consumption.

Studies have assessed the bioavailability of anthocyanins from whole blueberries and blueberry juice by analyzing plasma and urine concentrations [12,23,102]. The findings show that consumed anthocyanins are transformed into conjugated derivatives, such as glucuronidated, methylated, and sulfated forms, which circulate throughout the body and exert biological effects. These metabolites are generated through enzymatic changes in the intestine and liver and microbial metabolism in the colon, aided by enterohepatic recirculation. This recycling process prolongs the systemic presence of anthocyanin derivatives and may amplify their biological impact despite the low absorption of the original compounds.

Food matrices influence the stability and absorption of anthocyanins. Langer et al. [102] found that consuming whole blueberries led to higher plasma levels of anthocyanin metabolites compared to drinking blueberry juice. This suggests that dietary fibers, polyphenols, and other components in whole fruits may protect anthocyanins from degradation during digestion and enhance their absorption. Similarly, studies using isotopic labeling have elucidated the metabolic pathways of anthocyanins. Czank et al. [69] tracked ^13^C-labeled cyanidin-3-glucoside and discovered that many circulating compounds exist as metabolized derivatives rather than in their original form. This highlights that these metabolites may play a crucial role in the health benefits of anthocyanins.

Individual differences affect the bioavailability of anthocyanins in the human body. Variations in genetics, age, sex, gut microbiota composition, and diet affect the absorption, metabolism, and excretion of these compounds, causing significant differences in plasma levels between individuals [70,125]. The gut microbiota breaks down unabsorbed anthocyanins into smaller phenolic acids that are more easily absorbed and can have systemic effects while enhancing the local gut environment. The processing of blueberries through juicing, freeze-drying, or cooking can influence anthocyanin stability and matrix interactions, affecting bioavailability. Freeze-dried whole fruits might offer more stable anthocyanins and maintain interactions with fibers that encourage gradual release, whereas juices might lead to quicker but less prolonged absorption.

Pharmacokinetic studies have shown that anthocyanins are rapidly absorbed and eliminated, with peak blood levels occurring 1–3 h after ingestion, followed by rapid urinary excretion. Despite this rapid process, consistent consumption can sustain significant concentrations of anthocyanins and their metabolites, promoting prolonged health benefits [96,105]. These benefits likely result from the combined effects of native anthocyanins, their metabolites, and gut-produced phenolics, which contribute to antioxidant, anti-inflammatory, and cardiometabolic improvements in clinical studies. In conclusion, although native anthocyanins are minimally absorbed, factors such as metabolism, enterohepatic recycling, food matrix effects, and gut microbe interactions enhance the systemic presence of blueberry anthocyanins. These results emphasize the importance of consuming whole blueberries and their products, considering individual differences, and using metabolite-centered approaches to assess health benefits.

## 8. Factors Affecting the Bioavailability and Stability of Anthocyanins

The extent to which blueberry anthocyanins are absorbed and utilized by the body determines their biological effects and health benefits. Although these compounds are known for their antioxidant, anti-inflammatory, anticancer, and cardiometabolic benefits, they show low absorption rates and rapid metabolism. Understanding the factors affecting bioavailability is vital for enhancing dietary approaches and developing functional foods to improve their effectiveness. Bioavailability includes absorption in the digestive system, stability during digestion, metabolic changes, tissue distribution, and gut microbiota interactions.

**Figure 7:**
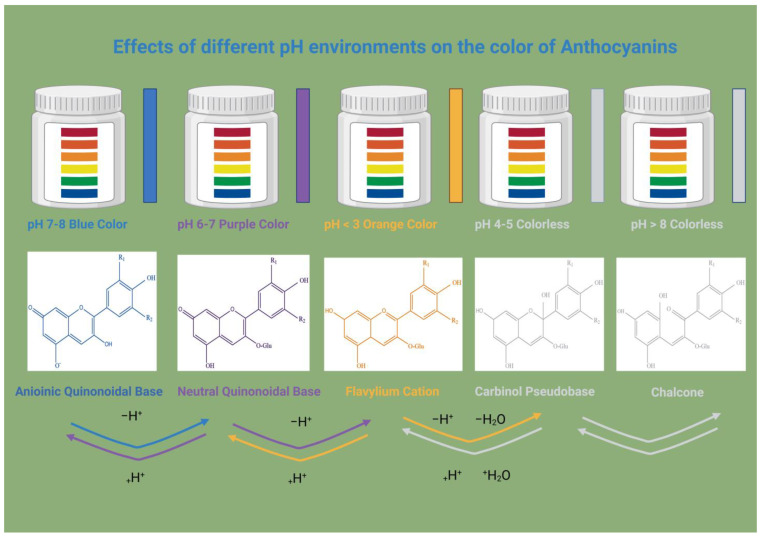
Effect of different pH levels on the stability and color of anthocyanins. The illustration was created using https://www.biorender.com/ (accessed on 22 February 2026).

The bioavailability and stability of blueberry anthocyanins are influenced by interactions between factors that affect their effectiveness as dietary bioactives. Blueberries have a distinct anthocyanin profile, and among their phenolic compounds, anthocyanins are vulnerable to environmental and processing conditions [104]. These compounds are chemically unstable and susceptible to degradation, leading to loss of color and bioactivity, and decreasing their bioavailability [126,127]. The chemical structures of anthocyanins, including hydroxylation, methoxylation, and glycosylation, determine their stability. The effect of pH on the color of anthocyanins has been shown in Figure 7.

External factors, such as solvent type, pH, temperature, light, oxygen, ionic strength, and the presence of metallic ions, proteins, or enzymes, can accelerate degradation. Anthocyanins are stable at acidic pH levels (2–3) but become unstable under neutral or alkaline conditions (pH 6–8), with instability increasing as the pH increases [128,129]. This sensitivity is due to the pyrylium ring, which can open to form a chalcone configuration, leading to chemical breakdown.

**Figure 8:**
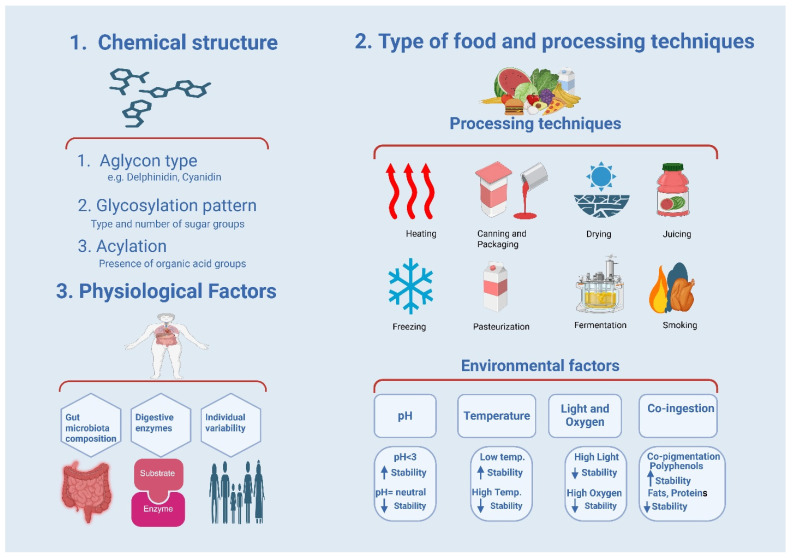
The effect of different factors, such as chemical structure, physiological factors, processing techniques, and environmental factors. The illustration was created using https://www.biorender.com/ (accessed on 22 February 2026).

Temperature is crucial; thermal treatments during processing can accelerate anthocyanin degradation, forming brown pigments in the presence of oxygen, and reducing visual appeal and biological function. Interactions with food matrix components, such as polysaccharides, proteins, and co-pigments, can influence anthocyanin stability by either protecting against or promoting degradation. Storage conditions, including light exposure and oxygen availability, affect degradation rates when they are uncontrolled. Understanding these influences is essential for optimizing anthocyanin retention in blueberry-based foods and products, as their stability affects their absorption and health-promoting potential [6]. Advanced strategies such as microencapsulation, pH stabilization, and co-pigmentation are being explored to preserve anthocyanin bioactivity during processing, storage, and digestion. Figure 8 describes the effect of different factors affecting the bioavailability of blueberry anthocyanins.

## 9. Enhancement of Stability and Bioavailability of Blueberry Anthocyanins

Anthocyanins are bioactive substances that confer antioxidant properties to blueberries and provide health benefits, including anti-inflammatory, heart-protective, brain-protective, and anticancer effects. Despite these benefits, their use in functional foods and nutraceuticals is restricted because of their low bioavailability, primarily due to their chemical instability in physiological and processing environments. These compounds are susceptible to environmental influences, such as pH, temperature, light, oxygen, and metal ions or enzymes, which can cause them to degrade, lose color, and reduce their biological effectiveness during storage, digestion, or processing [126,127]. Consequently, enhancing the stability and systemic availability of anthocyanins has become a major focus of research. Promising strategies include advanced delivery systems, such as nanoencapsulation, microencapsulation, and complexation with proteins or polysaccharides, which protect anthocyanins from degradation in the gastrointestinal tract and enable controlled release, better absorption, and improved interactions with the target tissues.

Protein–polyphenol complexes can create stable matrices that protect anthocyanins from oxidative stress and enzymatic breakdown, whereas nanocarriers enhance their solubility, permeability, and cellular uptake, thereby boosting their bioavailability [99]. Microencapsulation techniques using biopolymers, such as maltodextrin, chitosan, or modified starches, preserve anthocyanin content during thermal or acidic processing and allow targeted delivery to specific areas of the gastrointestinal tract, thereby increasing their microbiota-modulating effects and systemic bioactivity [28,101]. When anthocyanins are combined with other bioactive substances or food matrix components, their stability and absorption can be synergistically improved, broadening their functional food applications. These approaches provide solutions to address the instability and low bioavailability of blueberry anthocyanins, thereby enhancing their therapeutic potential in clinical and dietary settings.

### 9.1. Methodological Approach to Enhance the Bioavailability and Stability of the Anthocyanins

#### 9.1.1. Microencapsulation

Microencapsulation is designed to improve the stability and bioavailability of anthocyanins. This method, which involves encapsulating bioactive compounds within a carrier matrix, has been evaluated for its protective efficacy using standardized in vitro digestion models, such as INFOGEST, and for its release characteristics using kinetic models, such as Higuchi and Korsmeyer-Peppas. The evaluation was conducted using metrics such as encapsulation efficiency (EE%), stability under simulated gastric and intestinal conditions, and durability during storage [130,131].

Various encapsulating substances have been studied to stabilize blueberry anthocyanins. Carbohydrate-based materials, including maltodextrin, starch, carboxymethyl cellulose, Arabic gum, and xanthan gum, are used for their film-forming properties, affordability, biocompatibility, and controlled release of active ingredients. Polysaccharides protect anthocyanins by creating barriers against oxygen and light and maintaining suitable conditions during storage and gastrointestinal passage [132]. Protein-based carriers, such as gelatin, casein, and whey proteins, form stable complexes with anthocyanins through hydrogen bonding, hydrophobic interactions, and electrostatic interactions, preventing their degradation in acidic or neutral environments. Lipid-based carriers, such as lecithin and mono- or diglycerides, offer protection from enzymatic hydrolysis and improve solubility in water-based systems [133].

Spray drying is the primary method for encapsulating anthocyanins because of its simplicity, scalability, affordability, and low thermal degradation. This technique atomizes a solution containing anthocyanins and an encapsulating agent in a heated chamber, creating stable microcapsules through rapid solvent evaporation. The produced microparticles were evaluated for their moisture content, moisture absorption capacity, and flowability. Their effectiveness was confirmed by examining anthocyanin retention post-processing and stability during storage under specific humidity and temperature conditions. Importantly, their functionality was determined by analyzing release kinetics in simulated gastric (SGF) and intestinal (SIF) fluids, which often exhibited a delayed release pattern suitable for targeted delivery to the intestines [134,135]. This method transforms bioactive compounds into stable powders for integration into functional foods, drinks, and nutraceuticals. The effectiveness of spray drying depends on the choice of carrier, inlet and outlet temperatures, feed rate, and anthocyanin-to-encapsulant ratio. The combination of maltodextrin with gum arabic improves encapsulation efficiency, powder flow, and reduces hygroscopicity while preserving antioxidant properties [136,137].

Recent research has highlighted the use of microencapsulation techniques to protect blueberry anthocyanins from degradation. Cai et al. [138] employed a blend of carboxymethyl starch (CMS) and xanthan gum (XG) to encapsulate anthocyanins, demonstrating that these microcapsules preserved anthocyanins in the stomach and enabled their controlled release in the intestine. Similarly, microcapsules made from chitosan combined with cellulose nanocrystals or sodium tripolyphosphate offered improved stability of anthocyanins during storage and enhanced their release under simulated digestive conditions [139,140]. Chitosan serves as an advantageous carrier due to its biocompatibility, biodegradability, and mucoadhesive properties, which can boost anthocyanin absorption by extending the retention time in the small intestine.

Microencapsulation systems are often designed to utilize the pH gradient in the gastrointestinal tract for controlled release. Anthocyanins remain chemically stable in their flavylium cation form under the acidic conditions of the stomach (pH 1.5–3.5); they quickly degrade and transform into colorless chalcones at the neutral-to-alkaline pH levels of the small intestine [6,128]. Consequently, pH-sensitive carriers, such as CMS/XG, chitosan, and protein-polysaccharide composites, have been developed to shield anthocyanins from stomach acid and retain them within the carrier matrix during gastric passage. This strategy prevents early release and ensures that intact anthocyanins are delivered to the intestine, where absorption occurs. For instance, Liao et al. [109] showed that microcapsules made from whey protein and casein successfully inhibited anthocyanin release in a simulated gastric environment. As intestinal conditions change, the carriers dissolve or swell, allowing the release of anthocyanins into the intestinal lumen. A significant challenge is that anthocyanins become unstable once released into the intestinal environment, highlighting the need to optimize carrier composition and release timing to align anthocyanin release with absorption periods, thereby improving bioavailability.

Cyclodextrins have been used to stabilize and control the release of blueberry anthocyanins. These compounds form inclusion complexes with anthocyanins, protecting them from oxidation, light, and pH-induced degradation. According to Flores et al. and Su et al. [107,141], cyclodextrin microcapsules enable gradual anthocyanin release under simulated colonic conditions, enhancing their potential bioavailability and preserving their antioxidant properties. However, the researchers noted that if the stomach is crucial for anthocyanin absorption, delayed release might reduce bioavailability, highlighting the need to align release profiles with target absorption sites for optimal absorption in the small intestine.

The selection of encapsulating materials is vital for maintaining the antioxidant activity during storage. Gum Arabic microcapsules offer quick release for immediate antioxidant effects, whereas protein-based capsules enable gradual release for extended benefits [142]. The combination of polysaccharides and proteins enhances encapsulation efficiency, thermal stability, and controlled release properties, making them effective for anthocyanin delivery in functional foods. Microencapsulation safeguards anthocyanins during storage by minimizing degradation by light, oxygen, and heat, and enhances their physiological effects after consumption.

Microencapsulation affects the absorption and metabolism of anthocyanins in living organisms, including humans. Encapsulated anthocyanins resist degradation in the digestive system, allowing more to reach the small intestine or colon for absorption or interaction with the gut microbiota. Research has shown that microencapsulated anthocyanins may increase gut microbiota diversity, promoting beneficial bacteria such as *Bifidobacterium* and *Lactobacillus*, which convert anthocyanins into bioactive compounds [98]. This dual role of protecting anthocyanins while facilitating their interaction with the gut microbiota enhances the bio-efficacy of blueberry anthocyanins.

Challenges remain in the optimization of anthocyanin microencapsulation. The selection of an appropriate encapsulating material, particle size, wall-to-core ratio, and processing parameters is crucial for stability, controlled release, and absorption. The target site for absorption must be considered when designing microcapsules, as each gastrointestinal region presents unique challenges. Future studies should focus on developing multifunctional systems that integrate pH responsiveness, controlled release, and enhanced delivery to the microbiota to increase the effectiveness of anthocyanins in vivo.

Microencapsulation effectively improves the stability, bioavailability, and physiological effects of anthocyanins. Through careful material selection and process optimization, anthocyanins can be protected during storage and digestion while enhancing their release and interaction with the gut microbiota. Advancements in microencapsulation techniques show promise for developing functional foods that maximize the health benefits of blueberry anthocyanins.

#### 9.1.2. Nanoparticle Systems

Nanoencapsulation has become an effective method for enhancing the stability, gastrointestinal endurance, and bioavailability of blueberry anthocyanins, which are vulnerable to pH, temperature, oxygen, and enzymatic breakdown [126,127]. Nanoencapsulation offers a protective layer that guards anthocyanins against harsh environmental and physiological conditions, enabling their controlled release and absorption in specific areas of the gastrointestinal tract. This technique maintains the structural integrity of anthocyanins while enhancing their cellular uptake and bioactivity.

Polymeric nanoparticles have been investigated for anthocyanin delivery because of their compatibility with biological systems, biodegradability, and formulation flexibility. These nanoparticles can be created through polymerization methods, such as organic or aqueous emulsion polymerization, interfacial polymerization, or by utilizing pre-existing natural or synthetic polymers [25,143]. Chitosan-based nanoparticles are promising because of their mucoadhesive characteristics, which extend the intestinal retention time and improve absorption [126,127,144]. Encapsulating anthocyanins in chitosan, carboxymethyl chitosan, or chitosan/β-lactoglobulin complexes enhances their stability across storage temperatures and pH. Studies have shown that encapsulated anthocyanins achieve absorption rates of up to 40.1%, compared to 17.2% for non-encapsulated anthocyanins, demonstrating the effectiveness of nanoencapsulation in overcoming degradation and absorption barriers [26,127]. The formation of ionic and hydrogen bonds within the nanoparticle structure slows anthocyanin release in intestinal fluids, protecting it from enzymatic degradation and pH-related breakdown.

Nanocarriers made from proteins, such as whey protein, casein, and β-lactoglobulin, offer benefits for stabilizing anthocyanins. These proteins interact with anthocyanins through hydrophobic interactions and hydrogen bonds, providing protection during gastric digestion and allowing their controlled release into the small intestine [109,145]. Anthocyanins encapsulated in whey protein and casein resist release in stomach acid but dissolve in the neutral-to-slightly alkaline conditions of the small intestine, where absorption is most effective. Cyclodextrin inclusion complexes provide sustained release and protection against degradation, especially in the colon, where microbial metabolism converts anthocyanins into bioactive metabolites [107,141].

Nanoliposomes, consisting of phospholipid bilayers stabilized by cholesterol, offer a promising method for nanoencapsulation. They protect anthocyanins during storage and gastrointestinal passage, retaining approximately 72.8% of these compounds after simulated intestinal digestion compared to 52% for unencapsulated anthocyanins [111,146]. These nanoliposomes improve absorption by promoting paracellular and transcellular transport across the intestinal linings. However, their stability can be affected by intestinal enzymes and bile salts. To address this issue, hybrid systems combining polymeric nanoparticles with liposomal structures have been developed, merging polymer protection with liposome biomimetic features. Liposomes coated with chitosan offer increased stability, slower anthocyanin release, and enhanced intestinal delivery, thereby optimizing bioavailability [26,147]. Encapsulating anthocyanins in nanoparticles enhances the interactions with the gut microbiota. Upon reaching the colon in an encapsulated state, they are broken down by microbial communities, producing bioactive phenolic metabolites and fostering beneficial bacterial growth, such as *Bifidobacteria* and *Lactobacilli* [98,108]. This dual role of protection during upper gastrointestinal passage and colon delivery enhances systemic and local activities.

Nanoencapsulation enhances anthocyanin solubility and dispersibility, preventing clumping and settling in food products or dietary supplements. Better dispersibility facilitates greater interaction with intestinal epithelial cells, thereby boosting the absorption efficiency [108,148]. Food-grade nanocarriers, such as chitosan, benefit functional food applications through their safety, biological compatibility, and controlled release potential. The nanoencapsulation of blueberry anthocyanins addresses the challenges of stability, gastrointestinal degradation, and limited bioavailability. Polymeric nanoparticles, protein-based carriers, nanoliposomes, and hybrid systems create protective barriers, regulate release rates, improve intestinal absorption, and foster microbiota interactions. To maximize the effectiveness of anthocyanins in nutraceutical and functional food applications, optimizing nanocarrier composition, surface characteristics, and release profiles ensures that these bioactive compounds maintain potency and bioavailability during storage and digestion.

### 9.2. Delivery Systems

**Figure 9:**
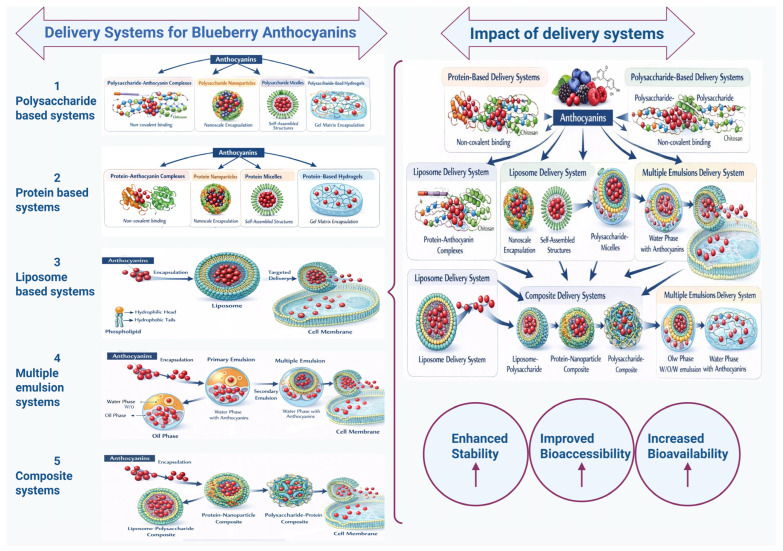
Different delivery systems and their impact on the enhancement of stability, improved bioaccessibility, and increased bioavailability. The illustration was created using https://www.biorender.com/ (accessed on 22 February 2026).

The natural instability of anthocyanins and their rapid breakdown in the digestive system restrict their use in foods, pharmaceuticals, and nutraceuticals. Stabilizing anthocyanin structures, minimizing degradation in the gastrointestinal tract, and boosting intestinal absorption are crucial challenges for their effective application [103]. Delivery systems have emerged as a promising approach to enhance the stability, controlled release, and bioavailability of anthocyanins by associating bioactive compounds with suitable carriers [149]. These systems adsorb, encapsulate, or chemically bind functional compounds to carrier matrices, addressing poor stability, low solubility, and limited bioaccessibility issues. They enable controlled release, extended residence time, and improved absorption by leveraging carrier properties and selective distribution [150]. They reduce dosages, minimize side effects, and maintain bioactivity during processing and digestion, making them suitable for sensitive compounds, such as anthocyanins. Microencapsulation is commonly employed for anthocyanin delivery, involving the trapping of bioactive compounds within protective wall materials to create microscale capsules that shield anthocyanins from environmental stressors such as light, oxygen, temperature, and pH. In microcapsules, anthocyanins serve as the core material, while polymers or biopolymers act as wall materials, determining the release behavior and protection efficiency [151]. Different categories of delivery systems are shown in Figure 9.

Recent research has focused on natural biopolymers, such as proteins, polysaccharides, and lipids, as anthocyanin carriers. These materials are used to construct microcapsules, nanoparticles, emulsions, and liposomes because of their biocompatibility, safety, and regulatory acceptance in food applications [152,153]. Protein-based systems facilitate anthocyanin binding through electrostatic and hydrophobic interactions, polysaccharide-based systems enhance stability through hydrogen bonding and steric protection, and lipid-based systems improve membrane permeability and cellular uptake of anthocyanins. The primary anthocyanin delivery systems include protein–anthocyanin, polysaccharide–anthocyanin, liposome-based, and composite delivery systems that combine multiple carriers for synergistic stabilization [153]. Composite systems have gained attention for integrating the functional advantages of different carriers and providing enhanced protection during digestion. The sustained release of anthocyanins from these systems is essential for maximizing their biological activity and bioavailability in vivo [154].

#### 9.2.1. Protein-Based Complexes

Protein complexes can enhance the stability and bioavailability of blueberry anthocyanins, as food matrices affect their stability. Protein- or fat-rich meals protect against anthocyanins, potentially improving their bioavailability. Among proteins, β-lactoglobulin has been extensively studied for its ability to stabilize anthocyanins under environmental and digestive conditions. However, certain anthocyanins, such as cyanidins, exhibit higher stability in food matrices and during pancreatic digestion than others [155,156]. Thus, identifying the optimal combinations of blueberry anthocyanins and proteins could guide the development of functional foods and nutraceuticals, improving health benefits and product formulation.

Lang et al. [106] explored the protective effects of α-casein and β-casein on blueberry anthocyanins using simulated gastrointestinal digestion models and animal experiments. Their research showed that α-casein enhanced anthocyanin stability, increasing the recovery rates of cyanidin 3-O-arabinoside and malvidin 3-O-arabinoside by 16.52% and 22.21%, respectively. The combination of proteins and anthocyanins led to elevated anthocyanin metabolite levels in rat plasma, suggesting that proteins stabilize anthocyanins during digestion and aid systemic absorption in vivo. While these findings are encouraging, it is important to consider interspecies variations, as rat digestion may not fully reflect human gastrointestinal (GI) physiology.

Whey protein significantly protects anthocyanins from degradation, according to Zang et al. [110]. Whey protein complexes reduce anthocyanin breakdown during simulated intestinal digestion while preserving the antioxidant properties. Their research showed that 26.46% of anthocyanins remained after an hour of intestinal digestion when combined with whey protein, compared to 12.37% of anthocyanins alone. Pan et al. [121] found that defatted soy protein powder enriched with blueberry polyphenols improved anthocyanin delivery in the TIM-1 digestion model, with 2.8 times more anthocyanins reaching the ileal efflux than with blueberry juice alone, suggesting enhanced potential for colonic microbial metabolism. These results indicate that protein-anthocyanin complexes reduce degradation in the gastrointestinal tract and enhance bioavailability and systemic effects.

Proteins such as α-casein and β-lactoglobulin are beneficial for functional foods because of their nutritional benefits, biodegradability, compatibility with biological systems, and resistance to pepsin digestion [157]. These proteins form stable complexes with anthocyanins, which may prolong the presence of anthocyanins in the gastrointestinal tract and enhance small intestinal absorption. However, stabilization might slow anthocyanin release in the stomach, potentially hindering absorption if the stomach is the main uptake site. Therefore, the target absorption site must be considered when developing protein-based delivery systems for oral administration. By optimizing protein-anthocyanin interactions, it is possible to maximize bioavailability, ensuring better stability during digestion and greater health benefits for the consumer.

Protein complexes offer advantages over microencapsulation, as they form natural interactions that are more compatible with food matrices and require less processing. Microencapsulation using carbohydrates, gums, or proteins is commonly employed to stabilize anthocyanins and facilitate their controlled release in the intestinal environment [132,158]. Nanoencapsulation has gained attention for safeguarding anthocyanins and improving their absorption by enhancing intestinal permeability and resistance to pH-induced degradation [126,159]. While nanoencapsulation offers precise control over particle size and surface properties, protein complexes provide a straightforward, food-compatible solution with potential health benefits from the protein itself. Both methods underscore the significance of targeted delivery, addressing anthocyanin instability in the small intestine, and optimizing bioavailability.

Protein complexation is a practical method for improving anthocyanin stability and bioavailability, and it works alongside other encapsulation methods. By selecting suitable proteins and optimizing the complexation conditions, it is feasible to develop functional foods that enhance anthocyanin delivery, maintain bioactivity during digestion, and maximize absorption. Ongoing research combining protein complexes with micro- and nanoencapsulation techniques could further refine anthocyanin delivery, offering immediate antioxidant effects and long-term health benefits to consumers.

#### 9.2.2. Polysaccharide-Based Delivery Systems

Polysaccharides, natural polymers of monosaccharide units linked by glycosidic bonds, are widely used as carriers in anthocyanin delivery systems because of their affordability, biodegradability, stability, and bioactivity. Common polysaccharides used for encapsulating anthocyanins include alginate, pectin, chitosan, cellulose, starch, guar gum, xanthan gum, carrageenan, and Arabic gum. The molecular structure and performance of these polysaccharides are influenced by their biological sources, extraction methods, and post-processing treatments, which affect their interactions with anthocyanins [160]. The interactions between anthocyanins and polysaccharides occur through non-covalent forces, such as electrostatic interactions, hydrogen bonding, and π–π stacking, which enhance their stability and functionality. Fu et al. [161] showed that purple potato anthocyanins form stable complexes with pectin, inulin, starch, and cellulose through electrostatic interactions, thereby improving their stability and antioxidant activities. Gum arabic stabilizes anthocyanins via hydrogen bonding, particularly under oxidative stress induced by ascorbic acid [134]. Lee et al. [162] found that berry anthocyanins complexed with fucoidan through π–π stacking and electrostatic interactions showed higher cellular permeability, plasma stability, and a 3.24-fold increase in bioavailability compared to free anthocyanins, with anti-inflammatory and anticancer effects. Polysaccharide–polysaccharide composite systems exhibit superior encapsulation performance.

Tan et al. [120] developed chitosan–chondroitin sulfate complexes that encapsulated anthocyanins with 88% efficiency while enhancing thermal and ascorbic acid stability. Micro- and nanoscale polysaccharide delivery systems improve anthocyanin protection and control its release. Alginate-based microcapsules created through electrostatic interactions and hydrogen bonding achieved an encapsulation efficiency of 84.2%and enhanced anthocyanin stability [116]. Zhao et al. [115] developed chitosan–pectin nanoparticles with sizes of 100–300 nm and 66.68% encapsulation efficiency, enabling controlled anthocyanin release and enhanced pro-apoptotic effects against tumor cells. Mehran et al. [163] prepared starch–maltodextrin microcapsules that improved antioxidant activity and thermal stability of anthocyanins; acetyl group dissociation in modified starch promoted intestinal release through intermolecular repulsion. Polysaccharide-based microcapsules and nanoparticles enhance anthocyanin stability, bioactivity, and color retention under thermal and gastrointestinal stresses. Current research focuses on multifunctional composite carriers that integrate polysaccharide delivery with co-pigmentation strategies to improve the stability and biological efficacy of anthocyanins.

#### 9.2.3. Liposome Delivery Systems

Liposome-based delivery systems enhance the bioavailability and therapeutic efficacy of bioactive compounds, such as anthocyanins. These systems consist of bilayer vesicles made of phospholipids or synthetic amphiphilic compounds with hydrophilic and hydrophobic regions that enable the encapsulation and transport of substances [117]. Their amphiphilic characteristics, biocompatibility, biodegradability, and non-toxic and non-immunogenic nature render them ideal for food and pharmaceutical applications. Various methods have been used to develop anthocyanin-loaded liposomes with optimized encapsulation efficiency, particle size, and release behavior. Chi et al. [111] used response surface methodology to refine nanoliposome preparation, achieving an average particle size of 53.01 nm and an anthocyanin retention rate of 85.6% at 25 °C. Zhao et al. [164] employed the supercritical CO_2_ technique to create liposome capsules with a particle size of 159 nm and an encapsulation efficiency of 50.6%. Sun et al. [117] combined ethanol injection with ultrasound to produce nanoliposomes, resulting in an embedding rate of 91.1% ± 1.7% and reduced particle size compared to unloaded liposomes, enhancing intestinal release of anthocyanins. These studies have shown that liposomes enhance anthocyanin stability, maintain bioactivity, and enable targeted release at absorption sites [111,117]. Solid lipid nanoparticles (SLN) and nanostructured lipid carriers (NLC) are advanced lipid-based systems for anthocyanin delivery. SLN offer greater physical stability and prolonged release profiles than traditional liposomes. By incorporating liquid lipids into SLN, NLC improves encapsulation efficiency, loading capacity, and controlled release at specific sites [113].

Ravanfar et al. [112] reported SLN prepared with palmitic acid, pluronic F127, lecithin, and span 85, achieving 89.2% ± 0.3% encapsulation and an average particle size of 455 nm ± 2 nm. Similarly, Pimentel-Moral et al. [118] showed that anthocyanin-loaded NLC from wood hibiscus achieved 84% ± 4% encapsulation efficiency and a particle size of 344 ± 12 nm, with analyses confirming enhanced stability through lipid–polyphenol interactions. Liposomes, SLN, and NLC are effective strategies for improving anthocyanin stability, bioactivity, and controlled release. While liposomes excel in immediate delivery and protection, SLN and NLC offer benefits in sustained release and higher encapsulation capacity. Future studies should focus on the in vivo behavior and gastrointestinal stability of SLN- and NLC-loaded anthocyanins to realize their potential in functional foods and nutraceutical applications.

#### 9.2.4. Delivery Systems Based on Multiple Emulsions

Multiple emulsions, also known as double or secondary emulsions, are complex systems in which a primary emulsion is dispersed within another continuous phase. Configurations include oil-in-water-in-oil (O/W/O), water-in-oil-in-water (W/O/W), and solid-in-oil-in-water (S/O/W) systems [135]. These structures enable the encapsulation of hydrophilic or lipophilic substances, shielding them from environmental stress while enabling controlled release, targeted delivery, and potential reduction in food ingredients [135,165]. Despite their benefits, multiple emulsions are thermodynamically and kinetically unstable, which limits their practical application in food systems [166]. Hydrocolloids, proteins, and polysaccharides are used as emulsion stabilizers to enhance stability. The stability of emulsion-based systems is influenced by interfacial phenomena. Aniya et al. [167] examined double emulsions by employing interfacial tension measurements and confocal laser scanning microscopy (CLSM) to observe droplet structures. Their findings indicated that the combination of xanthan gum with pea protein resulted in smaller droplet sizes (D3,23,2), an increase in zeta potential magnitude, and enhanced creaming stability during storage, thereby directly associating the formulation with quantifiable physical stability. Similarly, Kanha et al. [168] used gelatin and Arabic gum B to form double emulsions that slowed free fatty acid release and extended anthocyanin protection in the yogurt.

In spray-dried microcapsules, anthocyanin release in simulated gastric juice followed diffusion, whereas freeze-dried microcapsules released anthocyanins through erosion and wall disruption. Composite systems that combine co-pigmentation and carrier-based delivery improve anthocyanin stability and bioactivity. Kanha et al. [169] found that four types of such microcapsules enhanced antioxidant capacity, encapsulation efficiency, and thermal stability. The stability of double emulsions relies on primary and secondary emulsions and the emulsification mechanism. Pickering particles have emerged as effective stabilizers, replacing surfactants to prevent droplet coalescence by adsorbing at the oil–water interface, providing steric hindrance, and modifying the interfacial properties [150,170]. Pickering-stabilized multiple emulsions offer nutritional benefits, including reduced fat and salt content compared to traditional W/O emulsions, which aligns with modern dietary trends. By encapsulating bioactive compounds within the inner phase, these emulsions enhance stability and allow controlled release in the gastrointestinal tract, making them promising for anthocyanin delivery.

#### 9.2.5. Composite Delivery Systems

Composite delivery systems that incorporate multiple wall materials enhance anthocyanin stability and bioavailability. Single-wall materials often fail to provide adequate protection and controlled release of the drug. Composite carriers integrate complementary features to enhance encapsulation efficiency, stability, and performance in the gastrointestinal tract. Righi da Rosa et al. [171] showed that anthocyanin capsules made with composite wall materials achieved encapsulation efficiencies over 60% and controlled release in simulated gastric juice. Dumitrascu et al. [172] used soybean protein and maltodextrin in spray drying to embed anthocyanins, creating thin-film spherical droplets that enhanced stability and bioavailability. Wang et al. [114] employed chitosan hydrochloride, carboxymethyl chitosan, and whey protein isolate to create nanocomposites with an average particle size of 332.2 nm, a zeta potential of 23.65 mV, and a 60.7% encapsulation efficiency. The double-layer coating improved anthocyanin stability under high pH and intestinal conditions while maintaining its antioxidant activity. The combination of co-pigmentation and composite encapsulation enhances the stability of anthocyanins. Tan et al. [119] developed bovine serum albumin–chondroitin sulfate core–shell nanocapsules, in which protein-polysaccharide interactions increased anthocyanin encapsulation (54.6% compared to 47.4% for single-material systems) and system stability.

Pan et al. [121] created microcapsules with soy protein isolate and high-methyl pectin via spray drying, achieving superior controlled release, sustained antioxidant activity, and minimal degradation at 25 °C and 35 °C. Hydrogels provide a promising composite delivery platform owing to their three-dimensional network, water retention, and biocompatibility. Composite hydrogels combine mechanical strength, stimulus responsiveness, and high drug-loading capacity. Li et al. [173] prepared an anthocyanin/chitosan–salicylaldehyde hydrogel, enhancing thermal stability with pH-responsive release. Zhang et al. [151] combined emulsification, internal gel formation, and spray/freeze-drying to produce gels with high encapsulation efficiency, small particle size, and retention rates above 70% in simulated gastric fluid and 15% in intestinal fluids. Li et al. [174] reported a pectin/sodium alginate composite hydrogel system for improving the bioaccessibility of phycocyanin and increasing its bioavailability up to 83.03%, protected against the gastrointestinal conditions. Composite delivery systems address the limitations of single-material carriers by improving encapsulation efficiency, stability, and controlled release. Optimizing the wall material type, proportion, and interactions is crucial for maximizing bioavailability while preserving anthocyanin biological activity. Table 5 presents the quantitative comparison of anthocyanin delivery systems, which elaborates the formulation parameters, digestion conditions, and performance endpoints.

### 9.3. Future Challenges and Practical Considerations for Industrial Transition

The transition of advanced anthocyanin delivery systems from lab-scale models to commercially feasible products presents a range of technical, regulatory, and practical hurdles that need to be systematically addressed in future research. This section offers an evidence-based examination of the main factors for industrial applications, combining literature insights to assist researchers and industry participants.

#### 9.3.1. Safety Assessment and Regulatory Acceptability of Carrier Materials

The acceptance of carrier materials by regulatory bodies differs by region. In the United States, the FDA’s Generally Recognized as Safe (GRAS) status is essential for food-related uses, whereas in Europe, the European Food Safety Authority manages approvals, including those for Novel Foods [139,140]. Proteins such as whey protein isolate, casein, soy protein isolate, and gelatin have GRAS approval, but must include allergen labeling for milk, soy, and animal-derived ingredients [106,110]. Polysaccharides such as maltodextrin, gum arabic, alginate, pectin, and modified starch are widely accepted. Nonetheless, the trend towards clean labels has caused some consumers to resist processed ingredients [136,137]. Lipid-based carriers, such as lecithin and mono/diglycerides, are GRAS-approved, although soy lecithin requires allergen labeling [111,146]. Certain research-grade carrier systems face regulatory challenges. Chitosan does not have GRAS status in the US for food use and is not approved as a Novel Food in the EU, restricting its use to dietary supplements [126,127,139]. Cyclodextrins have limited GRAS approvals for specific uses, like flavor encapsulation, and need individual evaluation for anthocyanin delivery systems [107,141]. For carriers requiring Novel Food approval, a safety dossier must include physicochemical characterization, toxicological studies (genotoxicity, 90-day oral toxicity), allergenicity assessment, and human tolerance studies, with costs ranging from €500,000 to €1,000,000 over 2–4 years [139]. For food applications, it is advisable to prioritize established GRAS carriers such as maltodextrin, gum Arabic, modified starches, and approved proteins [132].

#### 9.3.2. Sensory Limitations and Consumer Acceptability

It is crucial that delivery systems do not negatively affect sensory attributes, as consumer acceptance is key to their market success [167,168]. Chitosan-based systems face notable sensory challenges, with consumer acceptance ratings ranging from 2.1 to 3.2 out of 5 in beverages due to bitterness and astringency; however, flavor masking can enhance these scores to between 3.8 and 4.2 out of 5 [126,127]. Protein-based systems receive acceptance ratings of 4.0–4.5 out of 5 in dairy products when added at less than 1%, but these scores decline to between 3.2 and 3.8 out of 5 when the addition exceeds 2%, due to the cooked or sulfurous flavors [109,110]. Liposomes achieve moderate acceptance ratings of 3.5–4.0 out of 5 in beverages, with the lecithin flavor becoming noticeable above 0.5% concentration [111,146]. Multiple emulsions are highly accepted, scoring between 4.2 and 4.7 out of 5 for reduced-fat products, owing to their enhanced creaminess [165,167]. Particle size plays a critical role in sensory perception. For clear beverages, particles must be smaller than 100 nm to prevent turbidity, limiting the choices to soluble protein complexes and nanoemulsions [101,122]. Cloudy beverages can handle particles up to 5 μm, making microcapsules and liposomes viable options [135,136]. In dairy products, particles should be less than 50 μm to avoid a grainy texture, with addition levels kept below 2% [168,169]. The tongue can detect particles larger than 30–50 μm, with graininess noticeable above 100 μm [132]. Capsule supplements circumvent most sensory limitations, making them the most flexible category of new carriers [112,113].

#### 9.3.3. Thermal Processing and Stability Constraints

Industrial thermal processing significantly affects anthocyanin retention. HTST pasteurization at 72 °C for 15 s preserves 65–75% of free anthocyanins, whereas microencapsulation with maltodextrin and gum arabic results in 82–90% retention [6,132,136]. Chitosan nanoparticles maintain 78–84% retention, although partial aggregation occurs [126,127]. Nanoliposomes achieve 70–75% retention, with 15–20% leakage due to bilayer fluidization [111,146]. Whey protein complexes retain 80–85% [109,110]. In contrast, LTLT pasteurization at 63 °C for 30 min only retains 45–55% of free anthocyanins [6,132]. UHT sterilization at 135 °C for 5 s leads to significant degradation: free anthocyanins retain just 25–35%, while encapsulated systems retain 45–60% with some wall rupture [6,132]. Therefore, products treated with UHT should be avoided for anthocyanin fortification. Spray drying at high inlet temperatures (160–180 °C) retains 80–94% due to rapid moisture evaporation [135,136,163]. Baking at 180 °C for 20–30 min retains less than 10% of free anthocyanins, whereas encapsulated systems retain 25–55% depending on the wall material [132]. Extrusion at 120–150 °C with high shear results in 15–25% retention of free anthocyanins and 30–40% retention of encapsulated forms [132,163]. Polysaccharide carriers remain stable up to 180 °C, making them suitable for pasteurization and baking [136,137]. Protein carriers denatured above 70 °C, restricting them to low-temperature processing [109,110]. Lipid carriers experience phase transitions at 40–60 °C, making them unsuitable for thermal processing without protection [111,146]. Composite systems enhance stability, with chitosan-coated liposomes showing improved stability at temperatures of up to 80 °C [26,147].

#### 9.3.4. The Requirements Related to Shelf-Life and Storage Stability

Commercial products typically require a shelf life of 12–24 months. When stored at 4 °C, free anthocyanins maintain 45–55% of their stability after 6 months and 25–35% after 12 months [6,132]. Microcapsules made with maltodextrin and gum arabic preserve 82–90% of their content at 12 months when humidity is controlled [136,137]. Chitosan nanoparticles retain 78–84% at 6 months, although partial aggregation occurred [126,127]. Nanoliposomes show 70–75% retention at 6 months, with a leakage rate of 10–15% [111,146]. At 25 °C, free anthocyanins hold 35–45% of their stability at 3 months and 15–25% at 6 months, which is not commercially viable [6,132]. Spray-dried microcapsules achieve 75–82% retention at 6 months and 65–72% at 12 months [136,137]. Gum arabic microcapsules maintain 78–85% at 6 months and 68–75% at 12 months [136,137]. CMS/XG composite microcapsules demonstrate 85–90% stability at 6 months due to their resistance to moisture [138]. Accelerated stability testing (40 °C/75% RH, 3 months) forecasts 24-month stability under ambient conditions. Free anthocyanins retain less than 10% of their content after 1 month [6,132]. Maltodextrin and gum arabic microcapsules retain 45–60% after 3 months, despite caking [136,137]. CMS/XG microcapsules retain 70–78%, indicating superior moisture resistance [138]. For commercial shelf life, the water activity must be below 0.3, and the packaging should provide an oxygen barrier and light protection. Spray-dried microcapsules can retain 70–80% at 24 months with appropriate packaging [136,137].

#### 9.3.5. Analytics and Specification Related to Quality-Control

A strong analytical method is essential for commercial production. Testing of raw materials should confirm the carrier’s identity using FTIR or NMR, ensure that the purity exceeds 95% through HPLC or GC, verify that heavy metals are below 10 ppm via ICP-MS, and adhere to microbial limits as per USP [132]. In-process controls necessitate that the particle size remains within ±10% of the target using laser diffraction or DLS [135,136], zeta potential stays within ±5 mV [126,127], encapsulation efficiency is over 80% with less than 5% batch variation using HPLC [135,136], and moisture content is under 5% with water activity below 0.3 [136,137]. Testing of the finished product involves determining the total anthocyanin content through the pH differential method (AOAC) to meet label claims within ±10% [132], analyzing the anthocyanin profile using HPLC-DAD or UPLC-MS/MS to match the reference fingerprint with peak areas within ±15% [38,50,97], and employing a stability-indicating HPLC method to separate parent compounds from degradation products, ensuring that individual impurities are under 2% and total impurities are below 5% [132]. Dissolution testing using the USP apparatus is used to establish performance specifications [135]. Antioxidant activity is measured using ORAC, DPPH, or FRAP as an indicator of bioactivity [29,45]. Stability monitoring involves accelerated conditions (40 °C/75% RH) showing more than 90% stability at 1 month and over 80% at 3 months, and long-term conditions (25 °C/60% RH) showing more than 80% stability at 12 months and over 70% at 24 months. Degradation products were identified using HPLC-MS/MS [132].

#### 9.3.6. Industrial Translation and an Integrated Decision Framework

Drawing from the evidence presented earlier, a comprehensive decision-making framework is employed to choose anthocyanin delivery systems, considering product specifications, processing conditions, shelf-life requirements, and regulatory limitations [131,135]. Clarity is a key factor in selecting a delivery system for beverages. Transparent drinks necessitate the use of soluble protein complexes or nanoemulsions with particles smaller than 100 nm, as larger particles lead to cloudiness [101,122]. Systems based on microparticles and chitosan should be avoided because of issues with light scattering and precipitation. For cloudy beverages, microcapsules under five micrometers or liposomes are suitable, provided particles exceeding ten micrometers are avoided to prevent sedimentation [135,136]. In dairy products where a smooth texture is desired, protein-based carriers and liposomes with particles under 50 µm are appropriate when used at levels below two percent [109,110,168]. Chitosan should be avoided because of its bitterness, and microcapsules larger than 100 μm can create a grainy texture detectable by consumers [126,127]. The detection threshold of the tongue for particulates is 30–50 µm, making particle size control crucial for consumer satisfaction [132].

For baked goods and extruded products that need to withstand temperatures up to 180 °C, only heat-stable microcapsules, such as those made from modified starch, should be used, with a retention rate of 25–55 percent being commercially acceptable [132,163]. Protein-based carriers denatured above 70 °C, and liposomes ruptured under these conditions, rendering them unsuitable for thermal processing [109,146]. In the case of supplements in capsule or tablet form, there is significant flexibility, as encapsulation can mask off-flavors, and higher costs are permissible [112,113]. All delivery systems are viable, with a focus on maximizing bioavailability and stability rather than sensory and cost considerations. This makes supplements an ideal starting point for new delivery systems that require regulatory approvals [139,140]. Processing requirements also influence this choice. Pasteurization at 72 °C for 15 s is compatible with most systems, achieving 70–90 percent anthocyanin retention, depending on the carrier material [6,132]. Ultra-high temperature treatment at 135 °C for 5 s should be avoided, as encapsulated systems retain only 45–60 percent of the initial anthocyanin content, which is generally unacceptable for commercial products. Products that do not undergo thermal treatment can utilize any delivery system, including microencapsulation.

Shelf-life requirements dictate the level of protection required for products. Items with a shelf life of less than six months can utilize free anthocyanins if refrigerated, although retaining only 45–55 percent after six months may not justify the expense of encapsulation [6,132]. For products needing 6 to 12 months of stability at room temperature, microencapsulation is necessary, with spray-dried systems retaining 65–80 percent [136,137]. Products that must remain stable for 12 to 24 months require optimized spray-dried microcapsules, with systems using carboxymethyl starch and xanthan gum performing best, retaining 85–90 percent at six months and 70–80 percent at 24 months [138]. For stability beyond 24 months, carboxymethyl starch, xanthan gum microcapsules, or frozen storage are required [132].

The regulatory jurisdiction limits the choice of carrier materials. In the United States and Canada, chitosan should be avoided in food applications because of its lack of GRAS status, with preferred carriers being those with established GRAS status, such as maltodextrin, gum Arabic, modified starches, and approved proteins [126,139]. In the European Union, carriers without a history of consumption require Novel Food approval, making approved food additives a safer option [139,140]. In Asia, regulations differ by country, necessitating local regulatory consultations before product launches [132]. This framework, which considers product type, processing conditions, shelf-life requirements, and regulatory jurisdiction, offers stakeholders evidence-based guidance for choosing anthocyanin delivery systems for commercial use. The framework highlights that no single delivery system is suitable for all applications; the selection should be based on specific product and market needs.

## 10. Conclusions

Blueberry anthocyanins are at the forefront of food bioactive research due to their wide range of health benefits, including antioxidant, anti-inflammatory, cardiometabolic, neuroprotective, and anticancer properties. This review consolidates recent progress in understanding the structural variety, biological roles, and key factors affecting the stability and bioavailability of blueberries, emphasizing their significance as strategic assets for the development of functional foods and nutraceuticals. A significant obstacle to the effectiveness of blueberry anthocyanins is their inherent chemical instability and low bioavailability. Growing evidence indicates that their physiological effects are not solely due to the intact parent compounds but are significantly influenced by phase II conjugates and metabolites derived from the gut microbiota, which offer enhanced stability and extended systemic activity. This necessitates a shift from traditional absorption-based evaluations to metabolite-focused and systems-level approaches that more accurately reflect in vivo functionality. From the perspective of food science and technology, delivery strategies are an emerging trend that addresses anthocyanin degradation and limited gastrointestinal stability. Protein–polyphenol complexes, polysaccharide-based carriers, micro- and nanoencapsulation systems, liposomes, multiple emulsions, and composite delivery platforms have shown promising improvements in the processing stability, controlled release, and intestinal availability of phenolic compounds. Importantly, the use of food-grade, scalable, and clean-label materials is in accordance with current industrial and regulatory standards. Future research should aim to translate these delivery systems into industrially feasible applications while maintaining sensory quality and cost-effectiveness. Well-designed human intervention studies that incorporate metabolomics, microbiome profiling, and pharmacokinetic modeling are crucial for elucidating dose–response relationships, individual variability, and long-term health outcomes. A greater focus should be placed on whole blueberry matrices and synergistic phytochemical interactions, which may provide superior functionality compared to isolated anthocyanins. In conclusion, blueberry anthocyanins represent an intersection of food chemistry, nutrition, and delivery technologies. Advances in stabilization strategies and metabolite-driven bioavailability assessment are redefining the roles of these compounds in health-oriented food systems. This review highlights key trends and research priorities that can support the rational design and successful translation of anthocyanin-enriched blueberry products into next-generation functional foods.

## Figures and Tables

**Table 1:** Evaluation of recent reviews on blueberry anthocyanins and the distinct contributions of the present review.

Review	Coverage Years	Structural Analysis	Health Benefits Covered	Mechanistic Depth	Bioavailability Focus	Delivery Systems Discussed	Human Evidence Synthesis	Quantitative Parameter Analysis	Industrial Translation	Unique Contributions/Limitations
Kalt et al. [12]	2015–2019	Moderate	CVD, diabetes, cognition, vision	Moderate (pathway discussion limited)	Moderate (general discussion)	None	Extensive (epidemiological and clinical trials)	No	No	Comprehensive human evidence synthesis; limited mechanistic and delivery system coverage
Yang et al. [29]	2010–2021	Extensive (structure–function relationships)	Antioxidant, anti-inflammatory, neuroprotective	High (molecular mechanisms)	Limited (brief overview)	None	Limited (primarily preclinical)	No	No	Excellent structural and functional analysis; no delivery system discussion
Herrera-Balandrano et al. [28]	2010–2020	Moderate	General health benefits	Moderate	Extensive (bioavailability enhancement strategies)	Microencapsulation, nanoencapsulation, protein complexes	Limited (brief mention)	Partial (qualitative)	Limited	Best prior coverage of delivery systems; lacks quantitative parameter synthesis
Silva et al. [17]	2000–2019	Limited	CVD, diabetes, obesity, cognition	Limited (epidemiological focus)	Limited	None	Extensive (systematic review of human studies)	No	No	Strong epidemiological evidence; minimal mechanistic or delivery system analysis
Wu et al. [30]	2010–2022	Moderate	Antioxidant, anti-inflammatory, anticancer, neuroprotective	High (signaling pathways)	Moderate (general discussion)	Limited (brief mention)	Moderate (some human studies)	No	No	Good mechanistic coverage; limited delivery system analysis
Wang et al. [23]	2015–2023	Moderate	General health benefits	Moderate	Moderate (stability and bioavailability factors)	Microencapsulation, nanoparticles	Limited	Partial (some parameters)	Moderate	Recent update on stability; lacks systematic parameter comparison
Ashique et al. [7]	2015–2023	Limited	Phytochemical and therapeutic applications	Moderate	Limited	None	Moderate	No	Limited	Broad therapeutic scope; limited depth on delivery systems
Present review	2018–2025	Extensive (six anthocyanidins, glycosylation patterns, structure–activity relationships)	Comprehensive (neural, inflammatory, cancer, ocular, cardiovascular, diabetic, obesity, antioxidant)	High (pathway-specific: NF-κB, Nrf2, MAPK, PI3K/Akt)	Comprehensive (ADME, phase II metabolism, microbial catabolites, biomarkers)	Proteins, polysaccharides, liposomes, SLN, NLC, multiple emulsions, composites, hydrogels	Extensive (systematic synthesis with critical appraisal)	Yes (particle size, zeta potential, EE%, release, in vivo relevance)	Extensive (scalability, regulatory, sensory, shelf-life challenges)	First review integrating quantitative delivery comparison, metabolite-focused bioavailability, industrial translation, and gap analysis

**Table 2:** The summarized blueberry anthocyanin health benefits with in vitro, animal, and human evidence.

Health Domain	In Vitro Evidence	Animal Evidence	Human Evidence	Overall Certainty	Key Gaps
Neuroprotection	Strong, multiple mechanisms (ROS reduction, AChE inhibition, autophagy) [52,55,56]	Moderate, improved memory in neurotoxicity models [52,53]	Limited, one RCT shows cognitive improvement [54]	Low to Moderate	Mechanisms not confirmed in humans; need trials with mechanistic biomarkers
Anti-inflammatory	Strong, NF-κB, MAPK pathway modulation [48,62]	Moderate, reduced arthritis symptoms, colitis [59,60]	Moderate, epidemiological associations; some RCTs show biomarker improvement [61,64]	Moderate	Specific pathway inhibition not confirmed at achievable human concentrations
Anticancer	Strong, apoptosis, proliferation inhibition (supra-physiological concentrations) [30,67,75]	Limited, tumor growth slowing in mice [72]	Very Limited, epidemiological associations only [17]	Low	Concentration gap (μM in vitro vs. nM in vivo); no human intervention trials
Eye health	Strong, RPE cell protection from oxidative/light damage [78,80]	Moderate, Nrf2 activation in diabetic rat model [79]	Very Limited, one RCT showed post-bleaching recovery but no improvement in night vision [81]	Low	Clinical significance unclear; need better outcome measures
Cardiovascular	Strong, antioxidant, anti-inflammatory mechanisms [83]	Strong, reduced atherosclerosis in ApoE^−^/^−^ mice [82]	Moderate, RCTs show biomarker improvement; epidemiological support [12]	Moderate to High	MiRNA mechanisms from animals need human confirmation
Antidiabetic	Moderate, enzyme inhibition, glucose uptake [17]	Moderate, improved glycemic control in rodent models [86,87]	Limited, one RCT shows improved cardiometabolic parameters [88]	Moderate	Mechanisms (AMPK, PPAR) not confirmed in humans
Anti-obesity	Moderate, adipocyte modulation [91,92]	Mixed, metabolic improvement often without weight loss [89,90]	Limited, metabolic biomarker improvement without consistent weight loss [7,86]	Low to Moderate	Need longer trials with body composition outcomes
Antioxidant	Strong, chemical assays, cell culture [29,45,95]	Moderate, reduced lipid peroxidation in animals [4]	Limited, increased plasma antioxidant capacity; mechanism debated [30]	Low	Direct radical scavenging unlikely in humans; metabolite effects need study

**Table 3:** Concentration gaps and physiological plausibility across health domains.

Health Domain	In Vitro Active Concentration (Parent Compounds)	In Vitro Active Concentration (Metabolites)	Human Achievable Plasma Concentration (Parent)	Human Achievable Plasma Concentration (Metabolites)	Fold-Gap (Parent)	Fold-Gap (Metabolites)	Plausible Mechanisms in Humans
Neuroprotection [28,52,54,69,70,95,97]	1–100 μM	0.5–10 μM	1–100 nM	0.1–2 μM	10–1000×	1–50× (overlap at lower end)	Metabolite-mediated; indirect (gut–brain axis); Nrf2 activation; chronic exposure
Anticancer [17,28,30,69,70,72,96,97,98]	10–300 μM	1–50 μM	1–100 nM	0.1–2 μM	100–3000×	1–500× (limited overlap)	Local GI effects (colonic lumen 100–500 μM); metabolite activity at high end; microbiota-mediated
Eye protection and health [28,69,70,78,79,81,95,97]	5–50 μM	0.5–5 μM	1–100 nM	0.1–1 μM (plasma); unknown in retina	50–500×	1–50× (tissue accumulation unknown)	Possible tissue accumulation; metabolite activity; Nrf2 activation
Cardiovascular health [12,28,69,70,82,84,97]	0.1–50 μM	0.1–5 μM	1–100 nM	0.1–2 μM	1–500×	1–10× (significant overlap)	Metabolite-mediated, most plausible; miRNA modulation; endothelial effects; chronic exposure
Antidiabetic [12,28,69,70,86,88,89,96,97]	1–200 μM	0.5–25 μM	1–100 nM	0.1–2 μM (plasma); 5–50 μM (portal vein)	10–2000×	1–50× (portal vein concentrations higher)	Local GI effects (enzyme inhibition); portal vein metabolite activity; microbiota-mediated (SCFAs)
Anti-obesity [7,28,69,70,89,91,93,96,97]	10–100 μM	1–10 μM	1–100 nM	0.1–2 μM	100–1000×	1–50× (limited overlap)	Microbiota-mediated (SCFAs); indirect anti-inflammatory; metabolite activity at high end
Antioxidant [28,29,30,69,70,94,95,96,97]	1–50 μM	0.5–5 μM	1–100 nM	0.1–2 μM	10–500×	1–10× (partial overlap)	Unlikely for direct scavenging; plausible for Nrf2 induction; local GI effects

**Table 4:** Evidence-weighted examples of formulation effects on anthocyanin metabolite profiles.

Delivery System	Carrier Materials	Key Metabolite Changes Observed	Proposed Mechanism	Analytical Method	Study Type	References
Protein Complexes	α-casein	↑ Methylated conjugates (peonidin-3-glucoside-glucuronide) by 22–35%; ↓ Sulfated derivatives	Modulation of intestinal COMT and UGT activity; delayed gastric release	LC-MS/MS (urine)	Rat in vivo	[106]
	Whey protein isolate	Prolonged Tmax for phase II conjugates; ↑ Cmax for glucuronidated metabolites	Slow release in the small intestine; protection from intestinal degradation	HPLC-DAD (plasma)	In vitro digestion + Caco-2	[109,110]
Polysaccharide Carriers	Cyclodextrin inclusion complexes	↑ Protocatechuic acid (2.5-fold) from cyanidin; ↑ Syringic acid from malvidin	Controlled colonic release; enhanced substrate availability for microbial β-glucosidases	GC-MS (in vitro fermentation)	In vitro fecal fermentation	[107]
	Chitosan-pectin nanoparticles	↑ Fecal phenylpropionic acids and phenylacetic acids	Targeted colonic delivery; modulation of gut microbiota composition	UPLC-QTOF-MS (feces)	Mouse in vivo	[98,108,115]
	Alginate microcapsules	Delayed appearance of phenolic acids; sustained release over 8–12 h	pH-sensitive release in intestine; protection from upper GI degradation	HPLC-MS/MS (plasma)	Rat in vivo	[116]
Lipid-Based Carriers	Nanoliposomes	↑ Intact anthocyanins in plasma (18–25%); ↓ Ratio of conjugates to parent	Enhanced absorption of intact glycosides via paracellular/transcellular transport	LC-MS/MS (plasma)	Rat in vivo	[111,117]
	SLN/NLC	Prolonged circulation of glucuronidated and sulfated metabolites; enterohepatic recirculation	Lymphatic uptake; protection from first-pass metabolism	HPLC-MS/MS (plasma, bile)	Rat in vivo	[112,113,118]
Composite Systems	Chitosan-HCl/CM-chitosan/WPI nanocomplex	Triphasic release: early phase II conjugates (1–2 h); late phenolic acids (6–8 h)	Multi-compartment targeting: stomach protection, intestinal release, colonic delivery	UPLC-MS/MS (plasma)	Rat in vivo	[114]
	BSA-chondroitin sulfate core–shell	↑ Co-pigmentation metabolites; enhanced stability of acylated anthocyanins	Protein-polysaccharide electrostatic interactions; protection from pH-induced degradation	HPLC-DAD (in vitro)	In vitro stability	[119,120]
	Soy protein isolate/high-methyl pectin	Sustained antioxidant metabolite activity; minimal degradation at 25 °C/35 °C	Synergistic protection from protein-polysaccharide matrix; controlled release	ORAC, FRAP, HPLC (in vitro)	In vitro release	[121]

COMT: Catechol-O-methyltransferase; UGT: UDP-glucuronosyltransferase; LC-MS/MS: Liquid chromatography–tandem mass spectrometry; UPLC-QTOF-MS: Ultra-performance liquid chromatography–quadrupole time-of-flight mass spectrometry; HPLC-DAD: High-performance liquid chromatography–diode array detection; GC-MS: Gas chromatography–mass spectrometry; SLN: Solid lipid nanoparticles; NLC: Nanostructured lipid carriers; BSA: Bovine serum albumin; CM-chitosan: Carboxymethyl chitosan; WPI: Whey protein isolate; Tmax: Time to maximum concentration; Cmax: Maximum concentration; GI: Gastrointestinal. ↑: Increased; ↓: Decreased.

**Table 5:** Quantitative comparison of anthocyanin delivery systems.

Delivery System	Carrier Materials	Particle Size (nm)	Zeta Potential (mV)	EE (%)	Digestion Parameters (pH/Time/Bile)	Gastric Release (%)	Intestinal Release (%)	Bioaccessibility Improvement	Permeability (Papp, ×10^−6^ cm/s)	In Vivo Exposure	Key Performance Endpoint Improved	References
Protein Complexes												
	α-casein, β-casein	N/A (soluble complex)	N/A	N/A	SGF: pH 1.2, 2 h; SIF: pH 6.8, 2 h; Bile: 10 mM	N/A	N/A	N/A	N/A	↑ plasma metabolites 22–35%	Plasma metabolite profile	[106]
	Whey protein isolate	150–250	−20 to −25	76.5	SGF: pH 2.0, 2 h (pepsin); SIF: pH 7.0, 2 h (pancreatin, 10 mM bile)	12%	88%	2.1× vs. free	N/A	N/A	Intestinal retention	[109,110]
	Defatted soy protein	5–25 µm	N/A	82.7	TIM-1 dynamic model: gastric (pH 2.0, 2 h), intestinal (pH 6.5, 4 h), bile (10 mM)	<10%	65%	2.8× ileal efflux vs. juice	N/A	N/A	Ileal delivery	[121]
Polysaccharide												
	Chitosan-pectin NPs	100–300	+25 to +35	66.7	SGF: pH 1.2, 2 h; SIF: pH 6.8, 4 h; Bile: 5 mM	5–8%	62%	1.9× vs. free	2.8 ± 0.3	N/A	Cellular uptake	[115]
	Alginate microcapsules	850–1200 µm	−18 to −25	84.2	SGF: pH 1.2, 2 h; SIF: pH 6.8, 4 h; Bile: 10 mM	<5%	72%	2.2× vs. free	N/A	↑ AUC 1.8× (rats)	Bioavailability	[116]
	Starch-maltodextrin	5–50 µm	N/A	78.3–92.1	SGF: pH 1.2, 2 h; SIF: pH 6.8, 4 h; Bile: 2 mM	8–12%	68–75%	1.7× vs. free	N/A	N/A	Thermal stability	[163]
	Fucoidan complexes	<200	−30 to −40	85.2	Plasma stability assay (37 °C, 24 h)	N/A	N/A	3.24× plasma stability	4.2 ± 0.5	N/A	Plasma stability	[162]
Liposomes												
	Nanoliposomes	53.0	−15.2	85.6	SGF: pH 1.2, 2 h; SIF: pH 6.8, 4 h; Bile: 10 mM	15–20%	72.8% (retention)	1.4× vs. free	3.6 ± 0.4	N/A	Intestinal retention	[111,117]
	SC-CO_2_ liposomes	159	N/A	50.6	Same as above	12%	68%	1.3× vs. free	N/A	N/A	GI stability	[164]
	Chitosan-coated liposomes	180–220	+30 to +45	72.8	Same as above	<8%	82%	1.8× vs. free	4.1 ± 0.3	N/A	Mucoadhesion	[26,147]
SLN/NLC												
	SLN (palmitic acid)	455 ± 2	−25.3	89.2	SGF: pH 1.2, 2 h; SIF: pH 6.8, 4 h; Bile: 10 mM	5%	45% (8 h sustained)	2.5× vs. free (AUC)	N/A	↑ AUC 2.5× (rats)	Sustained release	[112]
	NLC (wood hibiscus)	344 ± 12	−28.5	84 ± 4	Same as above	<5%	58% (12 h sustained)	2.8× vs. free (AUC)	N/A	↑ AUC 2.8× (rats)	Loading capacity + sustained release	[112,113,118]
Multiple Emulsions												
	W/O/W (xanthan + pea protein)	5–15 µm	−35 to −45	82.3	SGF: pH 1.2, 2 h; SIF: pH 6.8, 4 h; Bile: 10 mM	10%	70%	1.6× vs. free	N/A	N/A	Creaming stability	[167]
	W/O/W (gelatin + Arabic gum)	10–25 µm	N/A	76.8	Yogurt matrix (21 d, 4 °C)	N/A	65% (in yogurt)	N/A	N/A	N/A	Food matrix stability	[168,169]
Composite Systems												
	CMS/XG microcapsules	150–300 µm	N/A	84.5–91.2	SGF: pH 1.2, 2 h; SIF: pH 6.8, 4 h; Bile: 10 mM	<5%	78%	2.3× vs. free	N/A	N/A	Gastric protection + intestinal release	[138]
	Chitosan-HCl/CM-chitosan/WPI	332.2	+23.65	60.7	SGF: pH 1.2, 2 h; SIF: pH 6.8, 4 h; Bile: 10 mM	8%	71%	1.8× vs. free	N/A	Triphasic plasma profile (rats)	Multi-compartment targeting	[114]
	Chitosan-chondroitin sulfate	150–250	+35 to +45	88.0	Thermal stability (80 °C, 2 h); Ascorbic acid stability	N/A	N/A	N/A	N/A	N/A	Thermal + oxidative stability	[120]
	BSA-chondroitin sulfate core–shell	180–220	−30 to −38	54.6	SGF: pH 1.2, 2 h; SIF: pH 6.8, 4 h; Bile: 10 mM	<5%	63%	1.3× vs. single-material	N/A	N/A	System stability	[119]
Hydrogels												
	Chitosan-salicylaldehyde	500–1000 µm	N/A	73.5	pH-responsive release (pH 2.0 vs. 7.4)	<10% (pH 2.0)	>80% (pH 7.4)	N/A	N/A	N/A	pH-responsive release	[173]
	Pectin/sodium alginate	300–600 µm	N/A	83.0	SGF: pH 1.2, 2 h (<10% release); SIF: pH 6.8, 4 h (>70% release)	<10%	83.0%	2.4× vs. free	N/A	↑ bioavailability 83.0% (rats)	Bioaccessibility + bioavailability	[151,174]

EE: Encapsulation Efficiency; SGF: Simulated Gastric Fluid; SIF: Simulated Intestinal Fluid; NPs: Nanoparticles; SLN: Solid Lipid Nanoparticles; NLC: Nanostructured Lipid Carriers; CMS: Carboxymethyl Starch; XG: Xanthan Gum; WPI: Whey Protein Isolate; CM-chitosan: Carboxymethyl chitosan; BSA: Bovine Serum Albumin; AUC: Area Under the Curve (plasma concentration-time); Papp: Apparent permeability coefficient; N/A: Not Available/Not Applicable; TIM-1: TNO Intestinal Model-1; SC-CO_2_: Supercritical Carbon Dioxide. ↑: Increasing.

## Data Availability

Data is contained within the article or Supplementary Material.

## Supplementary Materials

The following supporting information can be downloaded at: https://www.mdpi.com/article/10.3390/molecules31050793/s1, Figure S1: PRISMA 2020 Flow Diagram of Study Selection Process; Table S1: The studies were organized into five primary categories, each reflecting their main thematic focus; Table S2: Characteristics of studies included in the review (n = 174); Table S3: Detailed database search strategy and results; Table S4: Detailed breakdown of full-text articles excluded (n = 358); Table S5: PRISMA 2020 Checklist.
