# Supplementary material for: Enhanced Bioavailability and Health Benefits of Blueberry Anthocyanins: An Updated Review on Mechanisms and Approaches
Source: Molecules. 2026 Feb 27;31(5):793. doi: 10.3390/molecules31050793 (PMC12985910; doi:10.3390/molecules31050793)
Supplement: Supplementary file 1 [file molecules-31-00793-s001.zip › molecules-4117805-supplementary.pdf]

1. Complete search strategies for all databases

("blueberry"[Title/Abstract] OR "blueberries"[Title/Abstract] OR "Vaccinium"[Title/Abstract] OR "Vaccinium corymbosum"[Title/Abstract] OR "Vaccinium angustifolium"[Title/Abstract] OR "Vaccinium ashei"[Title/Abstract]) AND ("anthocyanin*"[Title/Abstract] OR "anthocyanidin*"[Title/Abstract] OR "cyanidin"[Title/Abstract] OR "delphinidin"[Title/Abstract] OR "malvidin"[Title/Abstract] OR "petunidin"[Title/Abstract] OR "peonidin"[Title/Abstract]) AND ("bioavailability"[Title/Abstract] OR "absorption"[Title/Abstract] OR "metabolism"[Title/Abstract] OR "pharmacokinetics"[Title/Abstract] OR "ADME"[Title/Abstract] OR "stability"[Title/Abstract] OR "degradation"[Title/Abstract] OR "health benefit*"[Title/Abstract] OR "antioxidant"[Title/Abstract] OR "anti-inflammatory"[Title/Abstract] OR "neuroprotective"[Title/Abstract] OR "cardiovascular"[Title/Abstract] OR "anticancer"[Title/Abstract] OR "antidiabetic"[Title/Abstract] OR "obesity"[Title/Abstract] OR "delivery system*"[Title/Abstract] OR "encapsulation"[Title/Abstract] OR "microencapsulation"[Title/Abstract] OR "nanoencapsulation"[Title/Abstract] OR "nanoparticle*"[Title/Abstract] OR "liposome*"[Title/Abstract] OR "emulsion*"[Title/Abstract]) AND ("2018/01/01"[Date - Publication] : "2025/12/31"[Date - Publication])

Fig. S1. PRISMA 2020 Flow Diagram of Study Selection Process

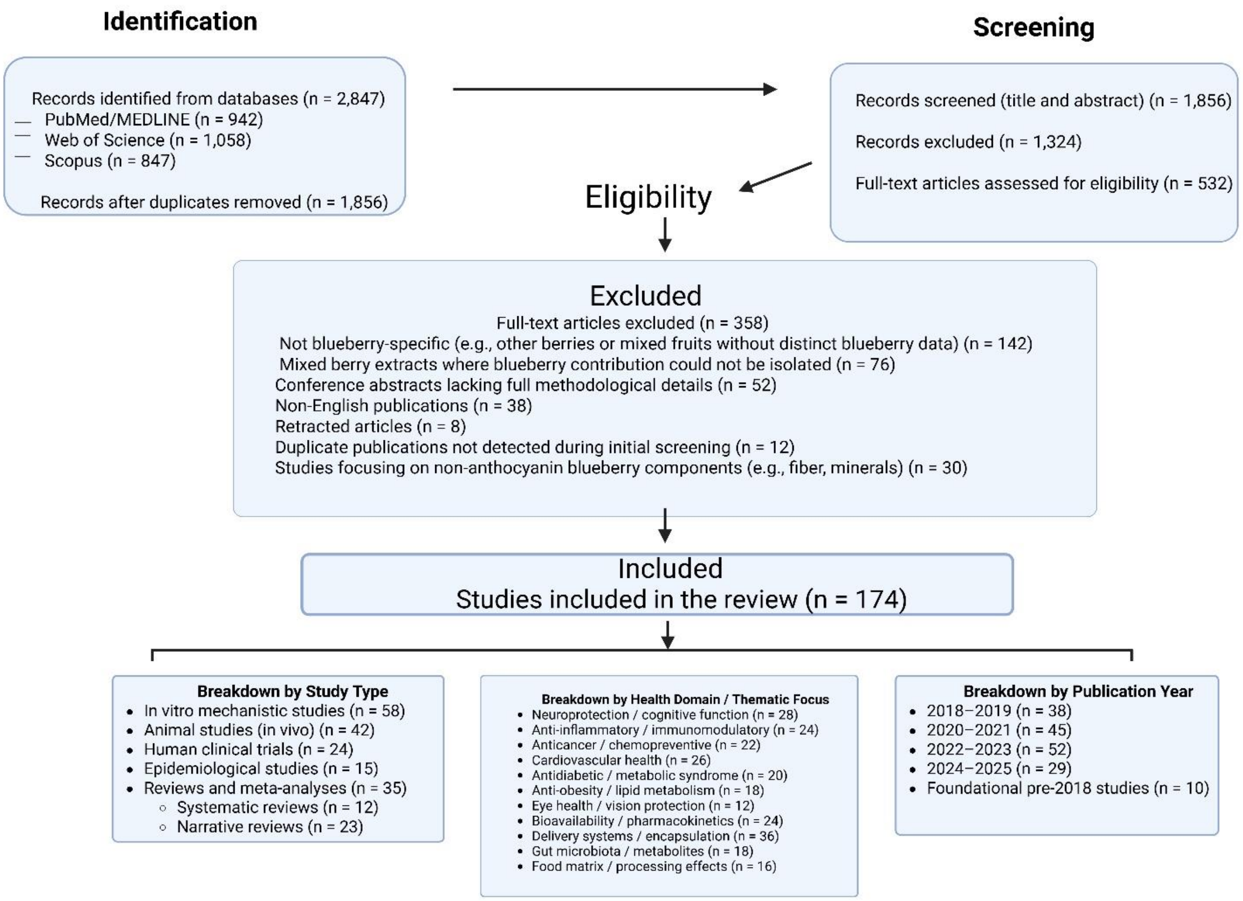

Thematic categorization of the studies

Table. S 1. The studies were organized into five primary categories, each reflecting their main thematic focus.

Category	Number of studies	Primary focus
In vitro mechanistic studies	68	Molecular pathways, cell culture models, enzyme assays
In vivo animal studies	47	Rodent models, tissue distribution, efficacy studies
Human clinical studies	28	Pharmacokinetics, intervention trials, epidemiological studies
Delivery system/formulation studies	26	Encapsulation, nanoparticles, liposomes, protein complexes
Foundational/contextual articles (pre-2018)	5	Seminal works for structural, mechanistic, or clinical context
**Total**	174	**--------**

Table S2. Characteristics of studies included in the review (n = 174)

Category	Subcategory	Number of Studies	Percentage (%)
**Study Type**	In vitro mechanistic studies	58	33.3%
	Animal studies (in vivo)	42	24.1%
	Human clinical trials	24	13.8%
	Epidemiological studies	15	8.6%
	Reviews and meta-analyses	35	20.1%
	• Systematic reviews	(12)	(6.9%)
	• Narrative reviews	(23)	(13.2%)
	**TOTAL**	**174**	**100%**
**Health Domain/ Thematic Focus***	Neuroprotection/cognitive function	28	16.1%
	Anti-inflammatory/immunomodulatory	24	13.8%
	Anticancer/chemopreventive	22	12.6%
	Cardiovascular health	26	14.9%
	Antidiabetic/metabolic syndrome	20	11.5%
	Anti-obesity/lipid metabolism	18	10.3%
	Eye health/vision protection	12	6.9%
	Bioavailability/pharmacokinetics	24	13.8%
	Delivery systems/encapsulation	36	20.7%
	Gut microbiota/metabolites	18	10.3%
	Food matrix/processing effects	16	9.2%
**Publication Year**	2018-2019	38	21.8%
	2020-2021	45	25.9%

	2022-2023	52	29.9%
	2024-2025	29	16.7%
	Foundational pre-2018 (selected)	10	5.7%
	**TOTAL**	**174**	**100%**
**Geographic Region**	North America (USA, Canada)	58	33.3%
	Europe (UK, Spain, Italy, etc.)	52	29.9%
	Asia (China, Japan, Korea, etc.)	48	27.6%
	Other (South America, Australia)	16	9.2%
**Blueberry Type**	Highbush (*V. corymbosum*)	82	47.1%
	Lowbush (*V. angustifolium*)	38	21.8%
	Rabbiteye (*V. ashei*)	24	13.8%
	Mixed/unspecified cultivars	30	17.2%

*Note: Studies may cover multiple health domains/thematic foci, so percentages exceed 100%.

Table S3. Detailed database search strategy and results

Database	Search String	Filters Applied	Records Retrieved
**PubMed/MEDLINE**	("blueberry"[Mesh] OR "blueberries"[Mesh] OR "Vaccinium"[Mesh] OR "Vaccinium corymbosum"[Mesh] OR "Vaccinium angustifolium"[Mesh]) AND ("anthocyanins"[Mesh] OR "anthocyanidin*"[All Fields] OR "cyanidin"[All Fields] OR "delphinidin"[All Fields] OR "malvidin"[All Fields] OR "petunidin"[All Fields] OR "peonidin"[All Fields]) AND ("bioavailability"[Mesh] OR "absorption"[Mesh] OR "metabolism"[Mesh] OR "pharmacokinetics"[Mesh] OR "stability"[All Fields] OR "health benefit*"[All Fields] OR "delivery system*"[All Fields] OR "encapsulation"[All Fields])	• English language<br>• 2018-2025<br>• Peer-reviewed<br>• Humans/animals	**942**
**Web of Science**	TS=(blueberry OR blueberries OR Vaccinium OR "Vaccinium corymbosum" OR "Vaccinium angustifolium" OR "Vaccinium ashei") AND TS=(anthocyanin* OR anthocyanidin* OR cyanidin OR delphinidin OR malvidin OR petunidin OR peonidin) AND TS=(bioavailability OR absorption OR metabolism OR pharmacokinetics OR stability OR "health benefit*" OR "delivery system*" OR encapsulation OR microencapsulation OR nanoencapsulation)	• English language<br>• 2018-2025<br>• Article or Review	**1,058**
**Scopus**	TITLE-ABS-KEY(blueberry OR blueberries OR vaccinium) AND TITLE-ABS-KEY(anthocyanin* OR anthocyanidin* OR cyanidin OR	• English language	**847**

	delphinidin OR malvidin) AND TITLE-ABS-KEY(bioavailability OR absorption OR metabolism OR "health benefit*" OR "delivery system*" OR encapsulation OR stability)*	• 2018-2025<br>• Article or Review<br>• Exclude conference papers	
		**TOTAL RECORDS**	**2,847**
		**After Duplicate Removal**	**1,856**

Table S4. Detailed breakdown of full-text articles excluded (n = 358)

Exclusion Category	Specific Reason	Number Excluded
**Not blueberry-specific (n = 142)**	Studies on strawberries, raspberries, and blackberries only	58
	Studies on mixed berries without distinct blueberry data	47
	Studies on other Vaccinium species (cranberries, bilberries) only	37
**Mixed berry extracts (n = 76)**	Berry blends where the blueberry contribution could not be quantified	48
	Commercial mixtures with unspecified composition	28
**Conference abstracts (n = 52)**	Insufficient methodological detail	31
	Preliminary data only	21
**Non-English publications (n = 38)**	Chinese	15
	Spanish	8
	German	6
	French	5
	Other languages	4
**Retracted articles (n = 8)**	Officially retracted from journals	8
**Duplicate publications (n = 12)**	The same data was published in multiple journals	8
	Pre-print plus published article (duplicate data)	4

**Non-anthocyanin focus (n = 30)**	Studies on blueberry fiber, minerals, and vitamins only	12
	Studies on other phenolics (chlorogenic acid, flavonols) without anthocyanin data	18
**TOTAL EXCLUDED**		**358**

Table S5. PRISMA 2020 Checklist

Section/Topic	Item #	Checklist Item	Location in Manuscript
**TITLE**			
Title	1	Identify the report as a systematic review.	Title page
**ABSTRACT**			
Abstract	2	See PRISMA 2020 for Abstracts checklist.	Abstract (page 1)
**INTRODUCTION**			
Rationale	3	Describe the rationale for the review in the context of existing knowledge.	Introduction (pages 2-5)
Objectives	4	Provide an explicit statement of the objective(s) or question(s) the review addresses.	Introduction (pages 4-5)
**METHODS**			
Eligibility criteria	5	Specify the inclusion and exclusion criteria for the review.	Section 2.3 (pages 10-11)
Information sources	6	Specify all databases, registers, websites, organisations, and dates of coverage.	Section 2.1 (page 9)
Search strategy	7	Present the full search strategies for all databases, including any filters and limits.	Section 2.1 (page 9)
Selection process	8	Specify the methods used to decide whether a study met the inclusion criteria.	Section 2.2 (page 10) + Figure S1 (Supplementary files)
Data collection process	9	Specify the methods used to collect data from reports.	Section 2.4 (page 11)
Data items	10a	List and define all outcomes for which data were sought.	Section 2.4 (page 11)

	10b	List and define all other variables for which data were sought.	Section 2.4 (page 11)
Study risk of bias assessment	11	Specify the methods used to assess risk of bias in the included studies.	Section 2.5 (page 11-12)
Effect measures	12	Specify for each outcome the effect measure(s) used in the synthesis.	Not applicable (narrative review)
Synthesis methods	13a	Describe the processes used to decide which studies were eligible for each synthesis.	Section 2.5 (page 11-12)
	13b	Describe any methods required to prepare the data for presentation or synthesis.	Section 2.5 (page 11-12)
	13c	Describe any methods used to tabulate or visually display results.	Section 2.5 (page 12) + Tables 1-5
	13d	Describe any methods used to synthesize results.	Section 2.5 (page 11-12)
	13e	Describe any methods used to explore possible causes of heterogeneity.	Section 2.5 (page 12)
	13f	Describe any sensitivity analyses conducted.	Not applicable
Reporting bias assessment	14	Describe any methods used to assess the risk of bias due to missing results.	Section 2.6 (page 12)
Certainty assessment	15	Describe any methods used to assess certainty in the body of evidence.	Section 2.5 (page 12) + Tables 2-3
**RESULTS**			
Study selection	16a	Describe the results of the search and selection process.	Figure S1 + Section 2.2

	16b	Cite studies that might appear to meet the inclusion criteria, but were excluded.	Table S4
Study characteristics	17	Cite each included study and present its characteristics.	Table S2 + References
Risk of bias in studies	18	Present assessments of risk of bias for each included study.	Section 2.5 (priority weighting)
Results of individual studies	19	For all outcomes, present for each study summary statistics.	Tables 2-5 throughout
Results of syntheses	20a	For each synthesis, briefly summarise the characteristics and risk of bias.	Section 2.5 + Tables 2-3
	20b	Present results of all statistical syntheses conducted.	Not applicable
	20c	Present results of any investigations of possible causes of heterogeneity.	Section 2.6 + Tables 2-3
	20d	Present results of any sensitivity analyses.	Not applicable
Reporting biases	21	Present assessments of risk of bias due to missing results.	Section 2.6 (page 12)
Certainty of evidence	22	Present assessments of certainty in the body of evidence.	Tables 2-3
**DISCUSSION**			
Discussion	23a	Provide a general interpretation of the results in the context of other evidence.	Sections 5-10
	23b	Discuss any limitations of the evidence included in the review.	Section 2.6 + Section 10.3
	23c	Discuss any limitations of the review processes used.	Section 2.6

	23d	Discuss implications for practice, policy, and future research.	Section 10.3 + Section 11
**OTHER INFORMATION**			
Registration and protocol	24a	Provide registration information for the review.	Section 2 (not registered)
	24b	Indicate where the review protocol can be accessed.	Not applicable
	24c	Describe and explain any amendments to information provided.	Not applicable
Support	25	Describe sources of financial or non-financial support.	Funding page (page 76)
Competing interests	26	Declare any competing interests of review authors.	Conflict of interest (page 76)
Availability of data	27	Report which of the following are publicly available: data, code, materials.	Available upon request
